# Viral Bcl-2-Mediated Evasion of Autophagy Aids Chronic Infection of γHerpesvirus 68

**DOI:** 10.1371/journal.ppat.1000609

**Published:** 2009-10-09

**Authors:** Xiaofei E, Seungmin Hwang, Soohwan Oh, Jong-Soo Lee, Joseph H. Jeong, Yousang Gwack, Timothy F. Kowalik, Ren Sun, Jae U. Jung, Chengyu Liang

**Affiliations:** 1 Department of Microbiology and Molecular Genetics and Tumor Virology Division, New England Primate Research Center, Harvard Medical School, Southborough, Massachusetts, United States of America; 2 Department of Molecular Genetics and Microbiology, University of Massachusetts Medical School, Worcester, Massachusetts, United States of America; 3 Department of Molecular & Medical Pharmacology, University of California, Los Angeles, California, United States of America; 4 Department of Molecular Microbiology and Immunology, University of Southern California, Los Angeles, California, United States of America; 5 Department of Physiology, University of California, Los Angeles, California, United States of America; University of Cambridge, United Kingdom

## Abstract

γ-herpesviruses (γHVs) have developed an interaction with their hosts wherein they establish a life-long persistent infection and are associated with the onset of various malignancies. One critical virulence factor involved in the persistency of murine γ-herpesvirus 68 (γHV68) is the viral homolog of the Bcl-2 protein (vBcl-2), which has been implicated to counteract both host apoptotic responses and autophagy pathway. However, the relative significance of the two activities of vBcl-2 in viral persistent infection has yet to be elucidated. Here, by characterizing a series of loss-of-function mutants of vBcl-2, we have distinguished the vBcl-2-mediated antagonism of autophagy from the vBcl-2-mediated inhibition of apoptosis in vitro and in vivo. A mutant γHV68 virus lacking the anti-autophagic activity of vBcl-2 demonstrates an impaired ability to maintain chronic infections in mice, whereas a mutant virus lacking the anti-apoptotic activity of vBcl-2 establishes chronic infections as efficiently as the wild-type virus but displays a compromised ability for ex vivo reactivation. Thus, the vBcl-2-mediated antagonism of host autophagy constitutes a novel mechanism by which γHVs confer persistent infections, further underscoring the importance of autophagy as a critical host determinant in the in vivo latency of γ-herpesviruses.

## Author Summary

Autophagy (‘*self-eating*’, lysosome-dependent degradation and recycling of the intracellular components in response to stress) and apoptosis (‘*self-killing*’, cells commit suicide in response to stress) are important host defense mechanisms against viral infections. γ-herpesvirus 68 (γHV68) encodes a Bcl-2 family protein, vBcl-2, that effectively antagonizes both autophagy and apoptosis and is required for chronic viral infection and pathogenesis. However, the relative contributions of the vBcl-2-mediated evasion of autophagy and apoptosis to γHV68 persistent infection remain largely unknown. Here, we characterized a series of vBcl-2 mutants to genetically and functionally distinguish these closely related activities of vBcl-2 in vitro and in vivo. We have found that the inhibition of autophagy by vBcl-2 is important for maintaining latent infections, while the anti-apoptotic activity of vBcl-2 is largely involved in efficient viral reactivation from latency. Our findings thus reveal a novel paradigm for the vBcl-2-mediated evasion of autophagy and apoptosis during chronic viral infection, identifying a vital role for autophagy in controlling γHV68 latent infection.

## Introduction

Apoptosis and autophagy, characterized by distinctive morphological and biochemical changes, are tightly regulated processes essential for homeostasis, development, and human diseases [1],[2]. Once triggered by internal inducers, such as DNA damage and viral replication, or by external stimuli, such as the engagement of the TNF receptor, apoptosis proceeds through a cascade of programmed internal proteolytic digestion, resulting in the collapse of cellular infrastructure, mitochondrial potential, genomic fidelity, and cell membrane integrity (for review see [1],[3]). Therefore, apoptosis represents an important effector of host immunity by eliminating virally-infected cells whose survival might otherwise prove harmful to the host [3]. In contrast to the self-destructing apoptotic program, cellular autophagy (Greek for ‘*self-eating*’) allows cells to engulf cytoplasmic materials, including long-lived proteins or aberrant organelles, into specialized double membrane-bound vesicles and deliver them to lysosomes for degradation and turnover (for review see [4],[5]). Although originally characterized as a cellular response to nutrient deprivation, autophagy has been increasingly recognized essential for protecting cells against pathogens [6]. Neuronal overexpression of the autophagy protein Beclin1 confers resistance to Sindbis virus infections [7]. Similarly, depletion of *beclin1* in plants aggravates the tobacco mosaic virus-induced hypersensitive response (HR) [8]. In addition to digesting cellular components, autophagy has been indicated to sequester virions and bacterial components for degradation [9],[10]. Thus, autophagy constitutes, in addition to apoptosis, an important host antiviral response [10]. However, the relative contributions and coordination of these two important pathways during viral infection remain largely unknown.

Yet as distinct as they are, the apoptotic and autophagic machinery converge at a number of points. One direct crosstalk between these two pathways is mediated in part by the functional and physical interaction of Beclin1, an essential autophagy activator, with Bcl-2, a prototype apoptosis inhibitor [11],[12]. Cellular Bcl-2 was originally discovered as an oncogenic protein in B-cell lymphomas, since then a number of proteins belonging to the Bcl-2 family have been identified, each possessing the signature of Bcl-2 homology domain (BH). The Bcl-2 family consists of both anti-apoptotic (e.g., Bcl-2, Bcl-X_L_, and Bcl-w) and pro-apoptotic (e.g., Bax, Bak, Bid, and Bad) members, which cooperate by forming homo- or hetero-dimers to regulate the cell's commitment to apoptosis [13]. A major mechanism by which the anti-apoptotic Bcl-2 proteins block apoptosis involves an extended hydrophobic groove on the surface of the proteins that serves as a binding-pocket for the α-helical BH3-domain of the pro-apoptotic Bcl-2 family proteins [14],[15]. Aside from its ability to interact with and inhibit pro-apoptotic family members like Bax and BH3-only proteins, the hydrophobic pocket of Bcl-2 also binds Beclin1 (the mammalian ortholog of yeast Atg6), which is part of a class III PI3 kinase complex required for the initiation of autophagosome membrane [16],[17]. In fact, the anti-autophagic action of Bcl-2 closely mirrors its capacity to bind and inhibit Beclin1 [12]. Intriguingly, structural analysis of Beclin1 revealed that it possesses a putative α-helical BH3 domain, which allows Beclin1 to dock into the hydrophobic pocket of Bcl-2. As such, Beclin1 is recently considered as a novel BH3-only protein [18],[19]. Although it remains unknown how Bcl-2 discriminates among its targets, the dual roles of Bcl-2 in apoptosis and autophagy suggest that a coordinated regulation may exist for Bcl-2 to conduct these two activities [20].

Given the important role of Bcl-2 in cell survival, many viruses have evolved to encode structural and functional orthologs of Bcl-2 (vBcl-2s) to prevent the premature death of the infected cells from sustained viral replication and associated diseases [21],[22]. All sequenced γ herpesviruses (γHV) encode a homolog of Bcl-2, including Epstein-Barr virus (EBV), Kaposi's sarcoma-associated herpesvirus (KSHV), herpesvirus saimiri (HVS), and the murine γ herpesvirus 68 (γHV68) [22],[23],[24]. The vBcl-2 of γHV68 (also referred to as M11) has been implicated in preventing Bax toxicity in yeast and blocking apoptosis in cultured cells when induced by diverse apoptotic stimuli [25],[26]. However, γHV68 vBcl-2 appears to be dispensable for acute infection in vivo and lytic replication in vitro, instead it is proved to be essential for efficient viral persistent replication as well as reactivation from latency, characteristic of all γHVs [25],[27]. Analysis of the three-dimensional structure reveals that vBcl-2 of γHV68 has limited sequence similarity to Bcl-2 but virtually adopts a fold similar to that of Bcl-2 [25],[28]. The seven-helix bundle (α1-7) of vBcl-2 forms a globular structure, where helices 2, 3, 4, and 5 define an extended hydrophobic surface cleft allowing vBcl-2 to associate with BH3 domains, especially those of Bak and Bax [25],[28]. Mutations within the BH3-binding groove abolished the ability of γHV68 vBcl-2 to interact with Bax and Bak, block apoptosis, and abrogate vBcl-2 function in persistent replication and reactivation from latency in vivo [25]. Thus, it is generally believed that vBcl-2 functions in vivo predominantly by binding and inhibiting pro-apoptotic Bcl-2 family proteins [25]. Yet, recent evidence favors a central role for vBcl-2s of γHV in blocking autophagy by directly interacting with Beclin1 via the BH3-binding groove of vBcl-2 [12],[19],[29]. Furthermore, the binding of purified vBcl-2 protein to the Beclin1-derived peptide appears to be the tightest when compared to peptides from the pro-apoptotic proteins, including BAX, BAK, BIM, PUMA, BID, and Noxa [28]. Unlike its cellular counterpart, this vBcl-2-Beclin1 complex can not be easily displaced by other BH3-only molecules, such as Bid or Bim [15],[28]. Accordingly, vBcl-2 exhibits an enhanced capacity for autophagy inhibition than cellular Bcl-2 [28],[30]. These findings raise the possibility that evasion of autophagy might also account for the biological effects of vBcl-2 in viral lifecycle and/or pathology. Nonetheless, due to the engagement of the hydrophobic surface groove of vBcl-2 by both the pro-autophagic BH3 domain of Beclin1 and the pro-apoptotic BH3 domain [28], mutations of vBcl-2 identified so far that disrupt Beclin1 binding and inhibition of autophagy also abolish its capacity to interact with BH3 peptides and inhibit apoptosis, adding to the complexity of genetically dissecting the in vivo role of vBcl-2-mediated autophagy inhibition and the vBcl-2-mediated antagonism of apoptosis in γHV68 infection.

In this study, we used loss of function mutagenesis to determine the role of vBcl-2-mediated anti-autophagy versus vBcl-2-mediated anti-apoptosis in the context of γHV68 infection. We found that a Beclin1-binding-deficient vBcl-2 mutant virus, which is impaired in autophagy inhibition but retains intact anti-apoptotic activity, was compromised in the maintenance of latency, though the initial viral establishment of latency was unaffected. In contrast, anti-apoptosis-defective vBcl-2 mutant virus infection was associated with a normal latent load but was largely impaired in efficient ex vivo reactivation from latency. Our findings thus demonstrate an as yet undefined function of autophagy in controlling viral infections. Unlike what was previously thought that anti-apoptosis features prominently the functions of vBcl-2 in vivo, our study reveals that an evasion of autophagy-mediated host innate immunity serves as a key aspect of γHV68 replication and pathogenesis.

## Results

### Minimal Region of vBcl-2 Required for Beclin1 Interaction in Yeast

**Figure 1:**
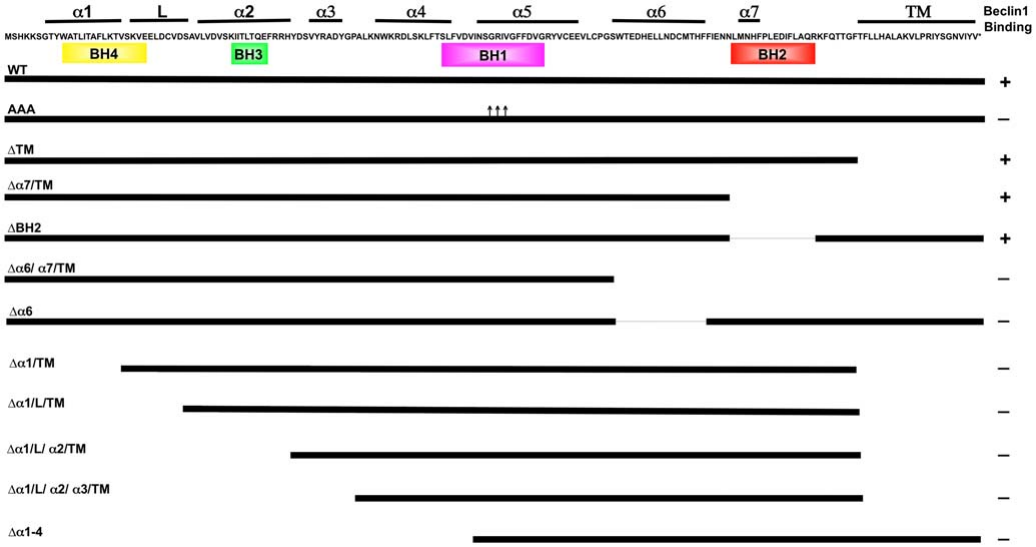
Schematic Representation of the Wild Type (WT) and Mutant vBcl-2 Constructs Interacting with Beclin1 in the Yeast-two-hybrid System. The 171-amino acid sequence of vBcl-2 is indicated at the top with the α-helical structures numbered according to previous publication [25]. Colored boxes denote the BH 1–4 domains in vBcl-2. “+” : positive interaction, “−” : no interaction. L: linker region; TM: transmembrane domain; Triple arrows denote the alanine substitutions at Ser85-Gly86-Arg87 within the BH1 domain of vBcl-2.

Beclin1 was originally identified as an interactor of Bcl-2 by a yeast two-hybrid screen [7]. The α-helical structure of the N-terminal region of Beclin1 (residues 88–150) mimics the BH3 domain of pro-apoptotic Bcl-2 family members, allowing it to associate with the hydrophobic BH3-binding groove on the surface of vBcl-2 [19],[28]. However, the Beclin1 peptide (*K_D_* 40 nM) binds to vBcl-2 with a much higher affinity than is observed for the Bak (*K_D_* 76 nM) and Bax peptides (*K_D_* 690 nM) [28], raising the possibility that Beclin1 may not necessarily share binding sites with the pro-apoptotic Bcl-2 family members for the hydrophobic groove of vBcl-2. To dissect the specific interacting domain of vBcl-2 for Beclin1 binding, we created a series of N- and C-terminal deletion mutations of vBcl-2 (Figure 1), and tested for their abilities to associate with the BH3-like domain (residue 88–150) of Beclin1 in the GAL4-based yeast two-hybrid assay [31]. Wild-type (WT) vBcl-2 readily interacted with Beclin1 BH3-like domain in the yeast two-hybrid assay. In contrast, a vBcl-2 mutant with a triple alanine substitution at the conserved residues of Ser85, Gly86, and Arg87 (hereafter termed as vBcl-2 AAA) within the BH3 binding groove that has been shown to abrogate BH3 peptide binding of vBcl-2, lost the ability to interact with Beclin1 (Figure 1). This was consistent with previous observations demonstrating that the hydrophobic groove of vBcl-2 is important for this function [25]. When screening our truncation mutants, we found that the C-terminal truncations of vBcl-2 up to α7 helix showed little to no effect on Beclin1 binding; yet a further truncation up to helix α6 (e.g. vBcl-2 Δα6/α7/TM mutant) severely impaired Beclin1 interaction in yeast (Figure 1), suggesting that the C-terminal boundary of vBcl-2 for Beclin1-binding lies within the α6 helix because no deletions from this end was tolerated. Of particular interest, a deletion of the BH2 domain only, which by analogy to the equivalent domain in Bcl-2 and Bcl-x_L_ has been shown to abolish binding and inhibition of Bax [32],[33], did not prevent vBcl-2 from binding Beclin1 in yeast (Figure 1). This data suggests that the BH2 region of vBcl-2 is dispensable for Beclin1 interaction. In contrast to the C-terminal moiety, removal of a small segment of the α1 helix at the N-terminus abrogated the ability of vBcl-2 to interact with Beclin1 (Figure 1). In fact, any segment truncation from the N-terminus of vBcl-2 resulted in the complete loss of Beclin1 binding (Figure 1). Thus, our results indicate that the minimal region required for Beclin1 interaction in yeast involves α helices 1-6 of vBcl-2. Although the N-terminal α1 helix is not part of the core hydrophobic α helices within the BH3-binding groove, it does appear to be critical for mediating Beclin1 interaction in yeast.

### vBcl-2 α1 Helix, but not the BH2 Domain, is Required for Beclin1 Interaction in Mammalian Cells

**Figure 2:**
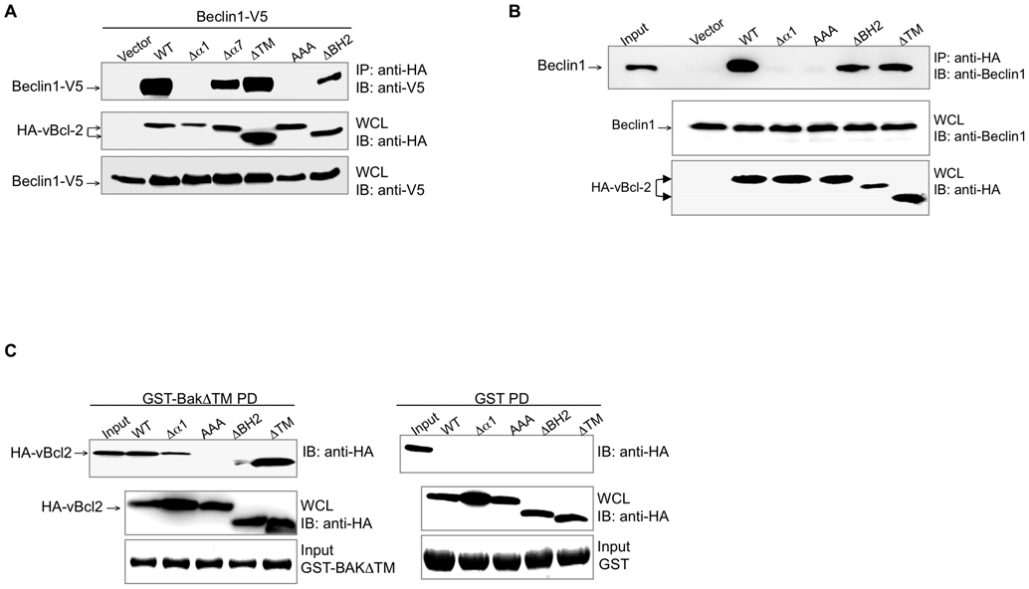
vBcl-2 Interaction with Beclin1. (A) Co-immunoprecipitation (Co-IP) of Beclin1 with WT or mutant vBcl-2. 293T cells were transiently transfected with the indicated constructs, followed by immunoprecipitation of HA-tagged vBcl-2 and immunoblotting of V5-tagged Beclin1. (B) Co-IP of WT or mutant vBcl-2 with endogenous Beclin1. 293T cells were transfected with the indicated vBcl-2 constructs, followed by immunoprecipitation of HA-tagged vBcl-2 and immunoblotting of endogenous Beclin1. 1% whole-cell lysates (WCLs) was used as the input. (C) vBcl-2 interaction with Bak protein. 293T cells were transfected with WT and mutant forms of vBcl-2 as indicated. At 48 h posttransfection, WCLs were mixed either with GST-BakΔTM fusion protein (left panel) or with GST alone (right panel) for an in vitro GST pull-down (GST PD) assays. GST fusion proteins used for the pulldown assay are indicated (bottom panel). 1% WCL was used as the input. Data are representative of at least three experiments yielding similar results.

We next confirmed our yeast two-hybrid results in mammalian cells. 293T cells were transfected with the WT or mutant forms of vBcl-2 and/or V5-tagged Beclin1, followed by co-immunoprecipitation (co-IP) assays. Consistent with the yeast two-hybrid binding data, deletion of the N-terminal α1 helix of vBcl-2 abolished Beclin1 binding, as also seen with the AAA mutant of vBcl-2 (Figure 2A). The loss of binding activity was not due to defects in protein expressions, since both the Δα1 and AAA vBcl-2 mutants were expressed at equivalent levels to WT in transfected cells (Figure 2A). Yet, deletion of the α7 or BH2, one of the central components of the vBcl-2 hydrophobic cleft, had no significant effect on the interaction between vBcl-2 and Beclin1, as was seen with the deletion mutation of the C-terminal hydrophobic ‘tail’ (ΔTM) (Figure 2A). Similar results were also observed with endogenous Beclin1 in 293T cells, in that removal of the α1 helix but not the BH2 domain abolished endogenous Beclin1 binding (Figure 2B). These data thus indicate that while the BH2 region is structurally important for assembling the hydrophobic core on the surface of vBcl-2, it is dispensable for vBcl-2-Beclin1 interaction, whereas the N-terminal α1 helix of vBcl-2 serves as a Beclin1-interacting domain (Figure 2B). These data are consistent with those collected from the yeast two-hybrid assay.

Although the N-terminal α1 helix deletion has been previously shown not affecting the overall folding of Bcl-2 family proteins [28],[34], it remains possible that the inability of the vBcl-2 Δα1 constructs to bind Beclin1 could reflect the loss of proper folding of the protein. To clarify this, we tested whether the mutants of vBcl-2 retain the ability to associate with other BH3-domain-containing molecules. Bak has been previously shown to have the highest affinity to vBcl-2 in vitro among the pro-apoptotic Bcl-2 proteins [28]. We then performed in vitro GST pull-down assay using the bacteria purified GST-fused Bak protein that was incubated with the cell lysates of 293T cells transfected with the HA-tagged WT or mutant forms of vBcl-2 (Figure 2C). The TM domain of Bak was removed (referred to as GST-BakΔTM) to increase its solubility in *E. coli*. In agreement with previous studies [25],[28], we observed that Bak was able to associate with the WT and ΔTM mutant of vBcl-2, but not with the vBcl-2 AAA mutant (Figure 2C). No interaction was detected between the vBcl-2 and purified GST alone, indicating that the vBcl-2-Bak interaction was specific (Figure 2C). Notably, we found that the Δα1 mutant that lacks Beclin1-binding retained its ability to interact with Bak, whereas the ΔBH2 mutant that was able to bind to Beclin1 failed to interact with Bak (Figure 2C). Thus, the loss-of-function phenotype of the vBcl-2 mutants for Beclin1 or Bak binding, particularly that of Δα1 and ΔBH2, is less likely due to misfolding of the mutant vBcl-2 protein, rather, it implies that the mechanisms of vBcl-2 for binding with Beclin1 and Bak involve distinct contact sites within the hydrophobic groove of vBcl-2.

### vBcl-2 α1 Helix, but not the BH2 domain, is Required for the Inhibition of Beclin1-mediated Autophagy

**Figure 3:**
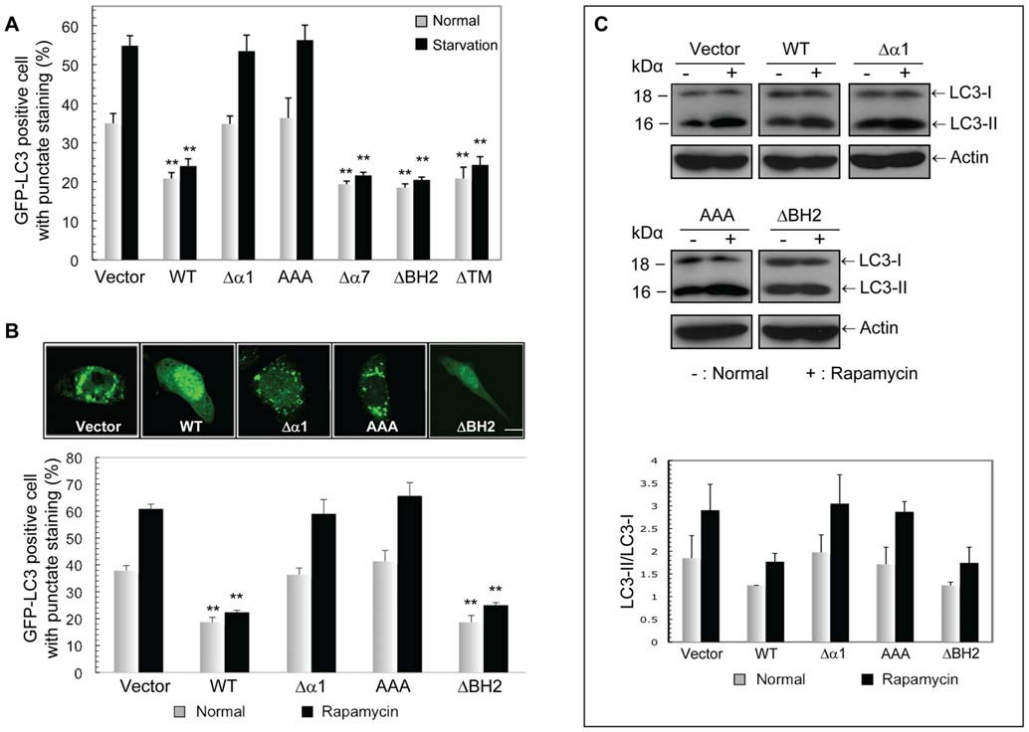
Anti-autophagic Activity of the vBcl-2 Mutant Proteins. (A) NIH3T3 cells stably expressing the WT and mutant forms of vBcl-2 were transfected with GFP-LC3. At 18 h posttransfection, cells were incubated under normal or starvation conditions for 4 h. Autophagy was quantified as mean±SEM of the combined results from three independent experiments. **, *P*<0.0001. (B) NIH3T3 cells expressing the WT or mutant forms of vBcl-2 as indicated were transfected with GFP-LC3 and treated with 2 µM rapamycin for 6 h. GFP-LC3 was detected using an inverted fluorescence microscope (top). Autophagy was quantified as mean±SEM of the combined results from three independent experiments. Scale bar, 5 µm; **, *P*<0.01. (C) NIH3T3 cells stably expressing the WT or mutant forms of vBcl-2, as indicated, were treated with 2 µM rapamycin. LC3-I and LC3-II levels were then determined by immunoblotting with an antibody against LC3 (top). Densitometric quantification of the LC3-II/LC3-I ratios under normal and rapamycin treatment conditions is shown at the bottom. Similar results were obtained from three independent experiments.

The interaction of Bcl-2 with Beclin1 largely correlates to its anti-autophagic activity [15]. We then assessed the effects of the vBcl-2 mutants binding to Beclin1, in particular that of Δα1 and ΔBH2 mutants, on Beclin1-dependent autophagy. NIH3T3 cells stably expressing empty vector (NIH3T3.Vector), WT vBcl-2 (NIH3T3.WT), or the mutant forms of vBcl-2, including the Δα1, AAA, Δα7, ΔBH2 and ΔTM mutants, were generated. To measure autophagy levels, we initially used the fluorescent autophagosome marker GFP-LC3 (a mammalian homologue of the yeast Atg8), which redistributes from a diffused cytosolic/nuclear staining to a punctate pattern in the cytoplasm upon autophagy stimulation [35]. We found that, consistent with their ability to co-IP Beclin1, the vBcl-2 ΔBH2, Δα7, and ΔTM mutants suppressed autophagy in these cells as effectively as WT did under nutrient depletion and rapamycin treatment, the established inducers of autophagy (Figure 3A and 3B). In contrast, the Δα1 mutant and AAA mutant of vBcl-2, which were unable to interact with Beclin1, failed to inhibit both starvation- and rapamycin-induced autophagy in the cells (Figure 3A and 3B). In accord, a significantly reduced number of autophagosomes per cell profile was observed in cells expressing WT and the ΔBH2 mutants, but not the Δα1 and AAA vBcl-2 mutants (Figure S1A). Immunoblotting was then performed with an antibody against LC3 to further measure autophagy in vBcl-2-expressing cells. During autophagosome formation, cytosolic LC3 (LC3-I) undergoes a covalent conjugation to phosphatidylethanolamine (PE) to yield a lipidated form of LC3, LC3-II, which displays higher electrophoretic mobility [36]. Consistent with the results of the GFP-LC3 puncta assay (Figure 3A and 3B), the conversion of LC3 from LC3-I to LC3-II was much reduced in WT and ΔBH2-expressing cells compared to that in Δα1- and AAA-expressing cells under normal and rapamycin treatment conditions (Figure 3C). It should be noted that the divergent features of the vBcl-2 mutant proteins in autophagy inhibition were not due to their differing protein expression since all tested mutants were expressed at levels equivalent to WT vBcl-2 in stably transfected cells (Figure S1B). Furthermore, all of the vBcl-2 mutants exhibited punctate cytoplasmic staining in the cells, similarly to the WT, except that the ΔTM mutant of vBcl-2 showed modest nuclear staining (Figure S1C). By analogy to the role of the equivalent region in Bcl-2 relatives, this hydrophobic ‘tail’ probably serves as a membrane anchor sequence in vBcl-2. While the C-terminal hydrophobic tail is not required for the function of vBcl-2, it may contribute to vBcl-2 by ensuring the proper subcellular localization of the protein. These data collectively demonstrate that the BH2 domain of the hydrophobic groove of vBcl-2 is not essential for suppressing Beclin1-mediated autophagy and its elimination does not affect Beclin1 binding, whereas the elimination of α1 helix leads to the loss of both Beclin1 binding and autophagy suppressing activity, reflecting a striking correlation between the ability of vBcl-2 to bind Beclin1 and its protection from Beclin1-mediated autophagy.

**Figure 4:**
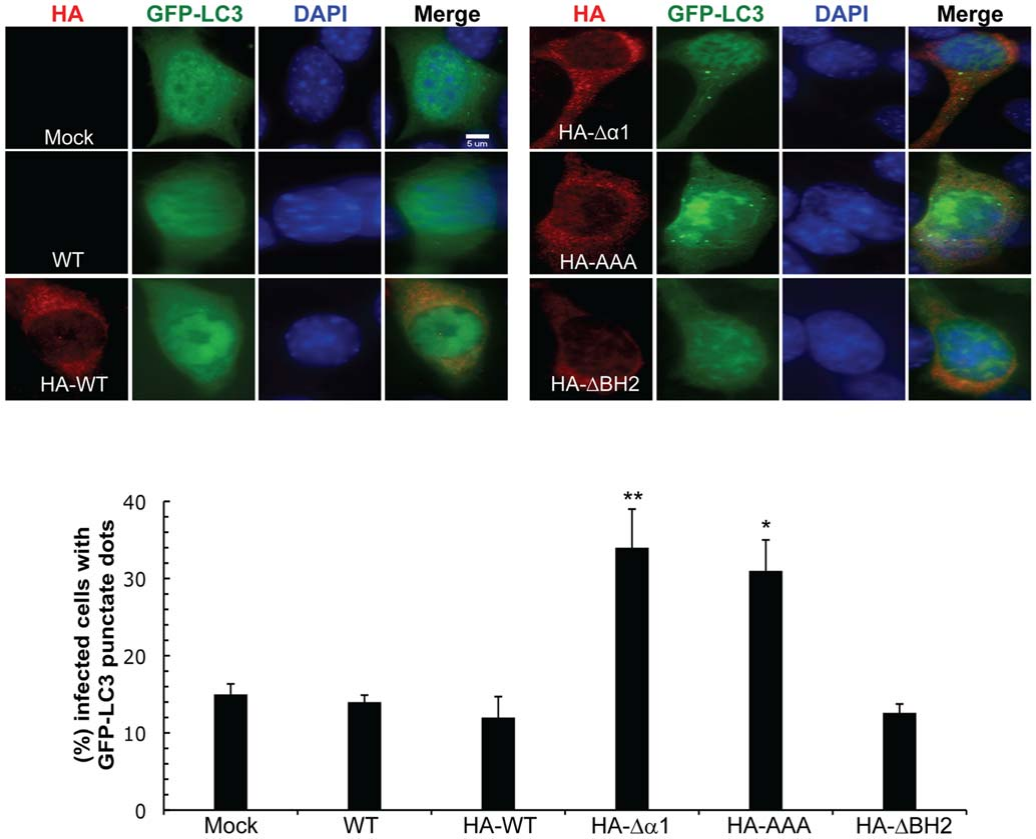
The Beclin1-binding α1 Helix of vBcl-2 is Required for Inhibition of Autophagy during γHV68 Infection. (top) Representative confocal images of GFP-LC3 and HA-tagged vBcl-2 (WT and mutants) in NIH3T3 cells infected with the indicated viruses (MOI = 5). Nuclei were counterstained with 4′,6′-diamidino-2-phenylindole (DAPI). Scale bar, 5 µm. (bottom) Quantification of the percentage of virally infected cells with GFP-LC3 punctate staining. Results shown represent mean±SEM of combined results from three independent experiments (200 cells per experimental condition). *, *P*<0.01 versus HA-WT; **, *P*<0.001 versus HA-WT.

To further address whether vBcl-2 inhibits Beclin1-mediated autophagy in virally infected cells, we generated recombinant γHV68 viruses that express HA-tagged WT (referred to as HA-WT) or mutant forms of vBcl-2 including AAA, Δα1 and ΔBH2 mutants (referred to as HA-AAA, HA-Δα1 and HA-ΔBH2, respectively) from its normal context in the viral genome, using the bacterial artificial chromosome (BAC) system (for detail please see Material and Methods). The genomic integrities of all recombinants were confirmed by restriction enzyme mapping and Southern blot analyses (Figure S1D). The vBcl-2 protein with the predicted molecular weight of 18 kDa was detected by immunoblotting using an anti-HA antibody with all of the recombinant γHV68 viruses in lytically infected 3T3 fibroblast cells (Figure S2A). Furthermore, the genetic manipulation of vBcl-2 did not affect expression of the neighboring v-cyclin (ORF72) in the recombinant viruses (Figure S2B). NIH3T3 cells were then infected with WT γHV68 or the recombinant γHV68 virus expressing HA-tagged WT vBcl-2 or its mutant derivatives. We found that cells infected with the recombinant γHV68 expressing HA-tagged WT vBcl-2 exhibited comparable levels of autophagy to those of WT γHV68-infected cells, suggesting that HA tagging does not affect vBcl-2 function (Figure 4). Notably, WT γHV68-infected cells exhibited levels of autophagy indistinguishable from those of mock-infected cells (Figure 4). In contrast, 3T3 cells infected with the Beclin1-binding-deficient vBcl-2 mutant viruses (γHV68 vBcl-2Δα1 and γHV68 vBcl-2AAA) showed significantly higher levels of autophagosome accumulation than those infected with the WT and ΔBH2 mutant viruses (Figure 4). These data indicate that γHV68 infection can trigger cellular autophagy, which is antagonized by vBcl-2 through Beclin1 inhibition. Taken together, vBcl-2 efficiently inhibits Beclin1-mediated autophagy in transfected and virally infected cells, and that this activity requires the α1 helix of vBcl-2, whereas the BH2 domain is dispensable for the anti-autophagic activity of vBcl-2.

### The BH2 Region, but not the α1 Helix, Is Required for vBcl-2 Anti-apoptotic Function

**Figure 5:**
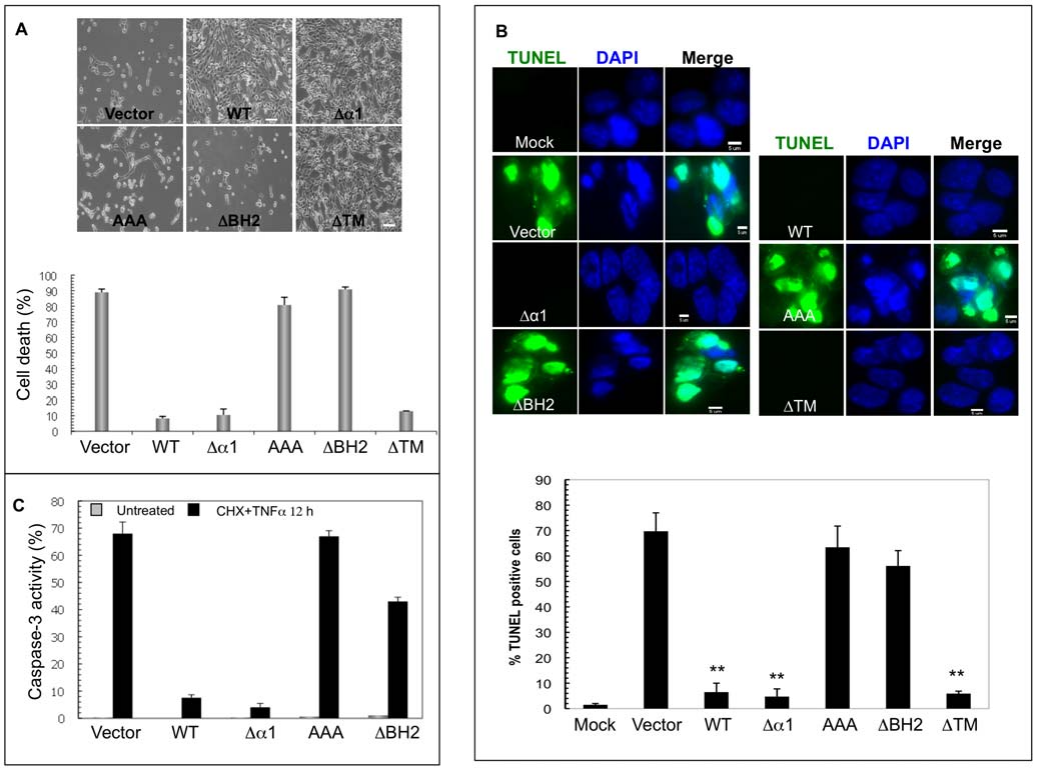
Anti-apoptotic Activities of the vBcl-2 Mutant Proteins. NIH3T3 cells stably expressing the WT or mutant forms of vBcl-2 were treated with TNFα and cycloheximide (CHX) for 12 h, then assayed for cell viability by trypan blue exclusion assay (A), or for apoptosis by TUNEL staining (B) or for the caspase 3 activation using flow cytometry (C). Apoptotic cells in (B) were counted under high power magnification (60×magnification). Mock, untreated condition. Data represents mean±SEM of combined results from three independent experiments. Scale bars, 100 µm (A), 5 µm (B). **, *P*<0.0001 versus vector cells.

Given the pivotal role of vBcl-2 in apoptosis inhibition, it is important to know if the regions of vBcl-2, which is required for binding and inhibiting Beclin1, are equally or differentially required for blocking apoptosis. To this end, we compared the abilities of WT or the vBcl-2 mutants to confer apoptosis resistance. Upon treatment with TNFα and cycloheximide (CHX) for 12 h, NIH3T3 cells stably expressing WT, ΔTM, or the Beclin1-binding deficient Δα1 vBcl-2 mutant survived significantly better than those expressing the empty vector, ΔBH2, or AAA vBcl-2 mutant (Figure 5A). Further quantification of the apoptotic cells via TUNEL staining revealed that the removal of the BH2 domain resulted in the failure of vBcl-2 in inhibiting apoptosis and the accumulation of apoptotic cells (Figure 5B). In contrast, deleting the α1 helix or the TM region did not affect the ability of vBcl-2 in suppressing apoptosis (Figure 5B). Equivalent results were obtained when we used propidium iodide (PI) staining to determine the accumulation of sub-G1 cells, which are considered to be apoptotic, via flow cytometry (Figure S3). Finally, the robust activation of caspase-3, an early event in apoptosis, was detected in cells stably expressing the vBcl-2 ΔBH2 and AAA mutants, whereas the expression of WT or the vBcl-2 Δα1 mutant significantly blocked caspase-3 activation elicited by TNFα/CHX- (Figure 5C). This further supports the notion that the deletion of the BH2 domain but not the α1 region seriously attenuates the ability of vBcl-2 to suppress caspase-dependent apoptosis. Taken alongside the autophagy analysis data, these results clearly demonstrate that the vBcl-2-mediated inhibition of apoptosis differs in important respects with its anti-autophagic activity. As summarized in Table 1, the deletion of the α1 helix in vBcl-2 that prevents Beclin1 binding and autophagy inhibition generally has little effects on vBcl-2' anti-apoptotic activity. In contrast, the removal of the BH2 region of vBcl-2 that abolishes vBcl-2's ability to block host-cell apoptosis has minimal effect on vBcl-2-mediated anti-autophagy. Thus, vBcl-2-mediated antagonism of autophagy can be structurally and functionally distinguished from its previously defined apoptosis inhibition activity, which then provides a general basis for evaluating their functional contributions in vivo during viral infections.

**Table 1:** Summary of the Anti-autophagic and Anti-apoptotic Activities of vBcl-2 mutants.

	Beclin1	Bak	Anti-Autophagy	Anti-Apoptosis
**WT**	+	+	+	+
**Δα1**	−	+	−	+
**AAA**	−	−	−	−
**ΔBH2**	+	−	+	−
**ΔTM**	+	+	+	+

### vBcl-2 Δα1 and ΔBH2 Mutants Have No Effect on γHV68 Lytic Replication in vitro and in vivo

**Figure 6:**
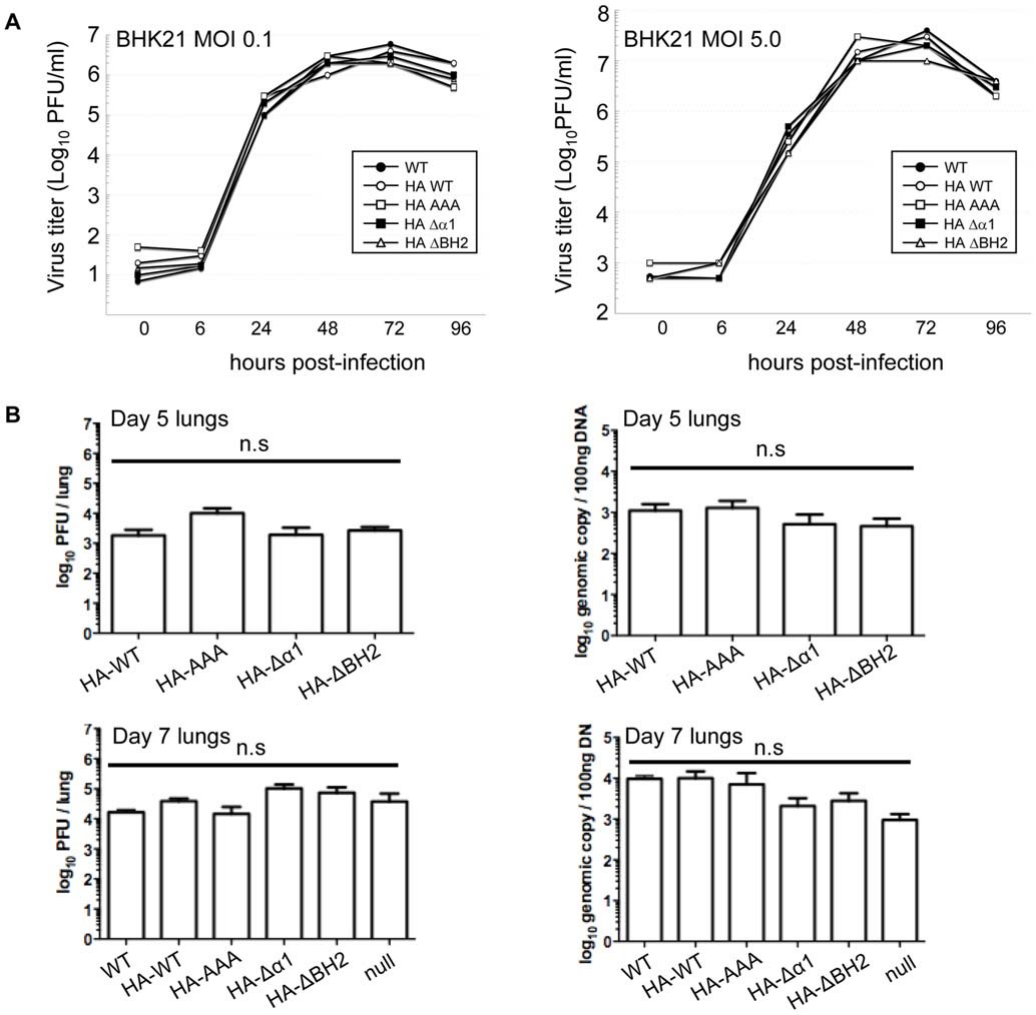
In Vitro and In Vivo Lytic Replication of the WT and Recombinant γHV68 Viruses. (A) Single-step (right) and multiple-step (left) growth curves of the WT and recombinant γHV68 viruses in BHK21 cells. (B) Acute replication of the WT and mutant vBcl-2 γHV68 viruses in the lungs of BALB/c mice at 5 dpi (up) and 7 dpi (down) after intranasal infection determined by viral titers in the lungs of the infected mice (left) or by real-time PCR of the viral genomic DNA (right). Mean±SEM of five mice per group/experiment. Data of 7 dpi is pooled from two separate experiments. The vBcl-2 Δα1 and ΔBH2 mutants did not yield significantly different results when compared to the WT in infectious virus titers [for Δα1, *P* = 0.82 (day 5); *P* = 0.08 (day 7); for ΔBH2, *P* = 0.49 (day 5); *P* = 0.54 (day 7); unpaired *t*-tests] and in viral genome loads in the lungs [for Δα1, *P* = 0.25 (day 5); *P* = 0.28 (day 7); for ΔBH2, *P* = 0.21 (day 5); *P* = 0.37 (day 7); unpaired *t*-tests]. n.s., not significant.

To determine the role of the vBcl-2-mediated inhibition of autophagy and apoptosis during viral infection, we first examined the in vitro growth properties of the recombinant γHV68 viruses expressing HA-tagged WT and the mutant forms of vBcl-2 in comparison to WT γHV68 in both BHK21 cells and NIH3T3 cells (Figure 6A and S4). The γHV68 vBcl-2Δα1 and ΔBH2 mutant viruses, as well as the vBcl-2AAA mutant, grew with the same kinetics as WT γHV68 in cultured cells (Figure 6A and S4), suggesting that the vBcl-2-mediated inhibition of autophagy and apoptosis are not required for lytic replication in vitro, which is consistent with the previous reports that γHV68 does not require vBcl-2 to replicate in vitro [25],[27],[37]. In accord with their growth in vitro in fibroblast cells, vBcl-2 mutant γHV68 viruses replicated at levels comparable to WT γHV68 in the lungs of intranasally infected BALB/c mice 5 or 7 days postinfection (dpi), as measured by plaque assay (Figure 6B, left panel) and real-time PCR (Figure 6B, right panel). No statistically significant differences were detected among γHV68 WT, the γHV68 mutant lacking vBcl-2 (vBcl-2-null), and the γHV68 recombinants expressing HA-tagged WT or mutant derivatives of vBcl-2 (Figure 6B). Independent isolates of γHV68 containing the Δα1 or the ΔBH2 mutations of vBcl-2 replicated normally in the lungs, arguing against the possibility of chance mutations having occurred elsewhere in the recombinant virus genomes (Figure S5A). These data collectively support a dispensable role for vBcl-2-mediated anti-autophagy and anti-apoptosis in viral lytic replication both in vitro and in vivo.

### The Beclin1-binding-deficient vBcl-2 Mutant Virus Is Impaired in Maintenance of Latency

**Figure 7:**
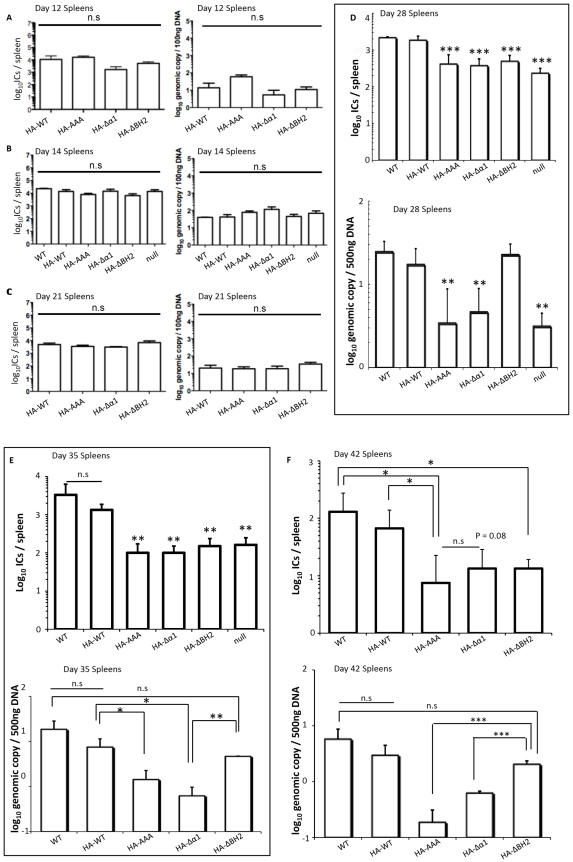
Distinct Roles for the vBcl-2-mediated Inhibition of Autophagy and Apoptosis in γHV68 Chronic Infections In Vivo. Splenic infectious centers measured at 12 dpi (A, left), 14 dpi (B, left), 21 dpi (C, left), 28 dpi (D, up), 35 dpi (E, up), or 42 dpi (F, up) and viral genome load measured by real-time PCR at 12 dpi (A, right), 14 dpi (B, right), 21 dpi (C, right), 28 dpi (D, down), 35 dpi (E, down), or 42 dpi (F, down), in the BALB/c mice intranasally infected with the WT or recombinant γHV68 mutants, as indicated (Mean±SEM of five mice per group/time point/experiment). Data of 14 dpi and 28 dpi is pooled from two and three separate experiments, respectively. Preformed infectious virus was negligible in all spleen samples. No significant difference was detected at 12 dpi (A), 14 dpi (B), and 21 dpi (C) with the Δα1 and ΔBH2 mutant viruses when compared to the WT in infectious center titers [for Δα1, *P* = 0.58 (day 12); *P* = 0.75 (day 14); *P* = 0.18 (day 21); for ΔBH2, *P* = 0.64 (day 12); *P* = 0.73 (day 14); *P* = 0.81 (day 21); unpaired *t*-tests] and in viral genome loads [for Δα1, *P* = 0.85 (day 12); *P* = 0.76 (day 14); *P* = 0.96 (day 21); for ΔBH2, *P* = 0.56 (day 12); *P* = 0.73 (day 14); *P* = 0.25 (day 21); unpaired *t*-tests]. At 28 dpi (D), 35 dpi (E), and 42 dpi (F), the decreased infectious center titers (top) of vBcl-2 mutant viruses, as compared to WT γHV68, were statistically significant [vBcl-2Δα1 versus vBcl-2AAA (42 dpi), *P* = 0.46; vBcl-2Δα1 versus WT γHV68 (42 dpi), *P* = 0.08; unpaired *t*-tests]. At 28 dpi (D), 35 dpi (E), and 42 dpi (F), the decreased viral DNA loads (bottom) of the vBcl-2Δα1, vBcl-2AAA, and vBcl-2 null mutants, as compared to WT γHV68 and the vBcl-2ΔBH2 mutant, were statistically significant as follows (unpaired *t*-tests): at day 28, vBcl-2Δα1 versus WT γHV68, *P*<0.01; vBcl-2AAA versus WT γHV68, *P*<0.01; vBcl-2-null versus WT γHV68, *P*<0.01; vBcl-2Δα1 versus vBcl-2ΔBH2, *P*<0.001; vBcl-2AAA versus vBcl-2ΔBH2, *P*<0.001; vBcl-2-null versus vBcl-2ΔBH2, *P*<0.001; at day 35, vBcl-2Δα1 versus WT γHV68, *P*<0.05; vBcl-2AAA versus WT γHV68, *P*<0.05; vBcl-2Δα1 versus vBcl-2ΔBH2, *P*<0.01; vBcl-2AAA versus vBcl-2ΔBH2, *P*<0.05; at day 42, vBcl-2Δα1 versus vBcl-2ΔBH2, *P*<0.001; vBcl-2AAA versus vBcl-2ΔBH2, *P*<0.001; vBcl-2ΔBH2 versus WT γHV68, *P* = 0.33. I.C., infectious center. *, *P*<0.05; **, *P*<0.01; ***, *P*<0.001.

After immune clearance of acute replication, γHV68 establishes latency in splenocytes, macrophages, and dendritic cells [38],[39],[40]. Disruption of vBcl-2 has been indicated to abrogate γHV68 from establishment of latency and/or reactivation [25],[27]. To determine which of the two activities of vBcl-2, anti-autophagy versus anti-apoptosis, might be primarily responsible for vBcl-2 function in vivo, we next evaluated the capacities of the recombinant γHV68 vBcl-2 mutant to confer chronic infection in mice in comparison to that of WT γHV68. BALB/c mice were intranasally infected with 5,000 PFU of γHV68 WT or mutants. By 12 and 14 days after infection, when WT γHV68 had reached its peak latent load in the spleen, the splenocytes were isolated and the viral latent loads were assessed by an infectious center assay as previously described [41]. Virus-driven splenomegalies were found in all infected mice, but no significant differences in spleen cell numbers were observable among the samples (data not shown). The virus titer of the vBcl-2 mutant including vBcl-2Δα1, vBcl-2ΔBH2, vBcl-2AAA, and vBcl-2 null, was similar to the WT in BALB/c mice (Figure 7A and 7B, left). Consistent with the infectious center data, the vBcl-2 mutant viruses showed peak levels of viral DNA loads comparable to the WT γHV68 at 12 and 14 dpi, suggesting the normal amplification of latent viruses in the spleen (Figure 7A right, 7B right). Similar observations could be made with the independently derived Δα1 and ΔBH2 mutant viruses (Figure S5B). Our results thus indicate that the loss-of-function mutations of vBcl-2 in autophagy or apoptosis inhibition, or both, have no appreciable impact on the establishment of viral latency in spleens, consistent with earlier finding that vBcl-2 is not required for the establishment of latency by γHV68 [37].

Given the lack of a role for vBcl-2 during early times of latent infection, we extended our analyses to determine whether vBcl-2-mediated autophagy and/or apoptosis affect viral maintenance of splenic latency at later time points. No significant difference in splenic latency between WT and the vBcl-2 mutant γHV68 was detected at day 21 (Figure 7C). However, by 28 days postinfection, the titers of vBcl-2 mutant viruses, including the independently derived vBcl-2Δα1 and vBcl-2ΔBH2 mutants, were dropped 6- to 10- fold when compared to the WT (*P*<0.001; Figure 7D up and S5C). Notably, this defect was not transient, but persisted 35 days and 42 days postinfection with a substantial, greater than 10-fold reduction in infectious center titers between WT and the vBcl-2 mutant γHV68, which represents an approximate 90% decrease in the frequency of latent viruses able to reactivate ex vivo. (Figure 7E and 7F up). These results indicate that there was a sustained deficiency of the vBcl-2 mutant viruses in the maintenance of latency after infection. A contraction of latently infected splenocytes was apparent at 42 dpi for both the WT and vBcl-2 mutant viruses (Figure 7F up). In all of the analyzed mice, preformed infectious viruses were undetectable in equivalent, freeze-thawed spleen samples (data not shown). Taken together, these data suggest that although vBcl-2 mutant viruses initially establish latency at levels equivalent to that of WT, there seems to be a steady decline of the latent virus reservoir at later time points in mice infected with the virus lacking a functional vBcl-2.

Since the infectious center assay does not distinguish between reductions in viral latent loads versus a failure of the latent virus itself to reactivate, we quantitated the viral genomes per splenocyte sample by real-time PCR to further measure the degree of viral latency. In agreement with the reduced infectious center titers at later time points, the viral genome load of the vBcl-2Δα1 mutant virus was severely reduced compared to that of WT viruses over the day 28 to day 42 time course in repeated experiments (Figure 7D, 7E, and 7F down, and S5D). In fact, the γHV68 vBcl-2Δα1 mutant, which expresses an anti-autophagy defective vBcl-2, was nearly as impaired as the AAA and vBcl-2-null mutant viruses (Fig. 7D, 7E, 7F down). The close correlation between viral genome loads and the frequency of latent γHV68vBcl-2Δα1 reactivation ex vivo at later times postinfection substantiates a severe latency defect of the γHV68 vBcl-2Δα1 virus. Since deletion of the α1 helix abolished vBcl-2's anti-autophagic activity but retained its anti-apoptotic function, the impaired latency associated with the vBcl-2Δα1 mutant virus infection at later times thus suggests that autophagy evasion by vBcl-2 plays an important role in maintaining γHV68 latent infection in splenocytes, whereas vBcl-2-mediated anti-apoptosis may not be absolutely required or sufficient for maintaining γHV68 latency. Indeed, the vBcl-2ΔBH2 mutant defective for apoptosis inhibition yet retaining Beclin1-binding and autophagy inhibition intact maintained viral genome loads equivalent to WT virus (Figure 7D, 7E, 7F down and S5D), arguing that the ΔBH2 mutation had no significant impact on viral latency. Yet, in marked contrast to the normal viral genome loads and splenocyte numbers (Figure S5E) in the ΔBH2 mutant virus-infected mice, the infectious center titer of the vBcl-2ΔBH2 mutant was significantly lower than WT γHV68 at later times, as previously described (Figure 7D, 7E, and 7F up). The disparity between the viral genome load and the latent viral titer argues that although the γHV68 vBcl-2ΔBH2 mutant virus is capable of maintaining a WT-level viral DNA load, it is unable to efficiently reactivate from latency in the spleen between day 28 and day 42 after infection. Since the BH2 domain is involved in the ability of vBcl-2 to inhibit apoptosis but not autophagy, this result thus suggests that the inhibition of host apoptosis by vBcl-2 is required for efficient ex vivo reactivation from the latent state particularly at later time points after infection, which is consistent with the previous report that the γHV68 vBcl-2 is required for latency reactivation [37].

The viral capacity of latency maintenance is prerequisite for establishing lifelong persistent infection of γHV and is often associated with various malignancies. Our study of the vBcl-2 mutant γHV68 viruses thus indicates that vBcl-2-mediated anti-autophagy and anti-apoptosis may play distinct role in γHV68 persistent infection, in that autophagy evasion by vBcl-2 is particularly required for the maintenance of viral latency, while the vBcl-2-mediated inhibition of apoptosis may play a role during upon viral reactivation.

## Discussion

Here we provide evidence that the vBcl-2-mediated Beclin1 binding and autophagy inhibition is necessary for the maintenance of γHV68 latent infection, whereas the capability of vBcl-2 to antagonize the host apoptosis response is required for efficient viral reactivation from latency ex vivo. Our study thus for the first time indicates that the vBcl-2-elicited anti-autophagy and anti-apoptosis activities are functionally and genetically distinct, also suggesting that the evasion of autophagy represents a critical step in the lifecycle and/or pathogenesis of γHVs.

The anti-apoptotic Bcl-2 proteins are structurally characterized by a hydrophobic surface groove that can accommodate the BH3 domain of the pro-apoptotic Bcl-2 family members as well as the BH3-like domain of Beclin1. Structural alignments of the BH3-like domain of Beclin1 and the BH3 domain of the pro-apoptotic Bcl-2 proteins revealed highly conserved topology and groove contact sites despite overall sequence variability, leading to the conclusion that Beclin1 is a putative BH3-only protein [15],[18],[19]. However, no apparent apoptosis induction activity has been found with Beclin1 in an in vivo context [15]. Moreover, our study indicates that despite their structural overlap, Beclin1 and pro-apoptotic Bcl-2 proteins interact with vBcl-2 through two discrete modes of binding that are dependent on a distinct region of vBcl-2. We show that removing the BH2 domain from vBcl-2 does not affect vBcl-2's capacity to bind and suppress Beclin1, but it significantly dampens its anti-apoptotic activity. By contrast, deleting the α1 helix does not affect vBcl-2's capacity to suppress apoptosis, yet strikingly impairs its anti-autophagic activity. We thus propose that the anti-apoptotic function of vBcl-2 is not required for its effect on autophagy inhibition and vice versa. Compared to a previous study of the vBcl-2-Beclin1 interaction in vitro [19], our in vivo data further demonstrates that mutations of vBcl-2 outside the vBcl-2-Beclin1 BH3 domain interface (e.g. vBcl-2Δα1) also affect vBcl-2-Beclin1 binding affinity, probably by altering the conformation of the hydrophobic cleft composed of the BH1, BH2, and BH3 domains. It can be speculated that not only do different BH3 domains have distinct binding footprints on the vBcl-2 surface groove as previously described [19], but that vBcl-2 undergoes different conformational changes when bound to distinct BH3 domain sequences. Thus, our data, in conjunction with recent findings [19],[28], provides a molecular explanation for the distinctly different roles of vBcl-2-mediated apoptosis and autophagy regulation in living cells.

γHV68 vBcl-2 is required for persistent viral replication and reactivation of the virus from latency [25],[27],[37]; two types of biological activities have been described: anti-apoptosis and anti-autophagy. However, it has not been possible to experimentally delineate their relative contributions to overall vBcl-2 functions in vivo. Our studies have allowed us to genetically distinguish the role of vBcl-2-mediated blockage of autophagy in vivo from vBcl-2-mediated anti-apoptosis by constructing a recombinant mutant virus that has the ability to block apoptosis but is unable to inhibit autophagy in infected cells. This mutant virus is highly attenuated in maintaining viral latency, suggesting that the vBcl-2-mediated inhibition of host-cell apoptosis is not sufficient to confer persistent infection, but rather that the vBcl-2-mediated blockage of Beclin1-dependent autophagy is required for the efficient maintenance of viral latency, a prerequisite for subsequent reactivation and transmission. This finding seems to be without precedent because vBcl-2 homologs have not been recognized to directly contribute to viral latent infection by interfering with the host autophagy machinery. Our data, however, do not rule out an important role for apoptosis, likely provided by other viral factors, in maintaining viral latency in vivo but, instead, they identify a vBcl-2-associated autophagy defect in chronic infection of γHV68. Analogous to γHV68 vBcl-2, KSHV-encoded vBcl-2 has been found to target Beclin1-dependent autophagy more strongly than cellular Bcl-2 [12],[30]. Given their poor overall amino acid homology to cellular Bcl-2 family members, the conservation of a mechanism of autophagy inhibition strongly supports the notion that interfering with the host autophagic machinery likely represents a common strategy for latent infection shared by these, and possibly other persistent γHVs. Nonetheless, it remains possible that the impaired latency that we observed with the vBcl-2 mutant γHV68 virus is not simply due to the disabled anti-autophagic activity of vBcl-2 but other as-of-yet undefined mechanisms. Future studies of viral replication and pathogenesis in mice lacking functional autophagy genes should help address this contingency.

Notably, two previous studies have also demonstrated the functional role of vBcl-2 during γHV68 chronic infection but with slightly different results [37]. In contrast to our findings that vBcl-2 is required for efficient maintenance of γHV68 latency, Gangappa *et al.* did not observe evident defects in the splenic latency of the vBcl-2 mutant [37]. As previously noted [27],[42], this discrepancy is potentially due to the use of different viral administration routes: intraperitoneal inoculation in Gangappa *et al.* versus intranasal inoculation in our study. On the other hand, de Lima *et al.* observed a reduced efficiency in the initial establishment of γHV68 splenic latency in the absence of a functional vBcl-2 as early as day 14 postinfection, which was subsequently recovered 6 months postinfection [27]. However, in our study with the vBcl-2 mutant viruses, the latency defect was not detected until 4∼6 weeks postinfection, the period in which a contraction of latently infected splenocytes was apparently observed. The basis for the differences between these data and our findings is not yet clear. It is possible that the higher dose inoculation (2×10^4^ PFU) used by de Lima *et al* might provoke stronger proinflammatory responses in the lung, which may presumably affect the initial viral seeding and amplification in the spleen. Alternatively, the vBcl-2 may carry out additional activities other than anti-apoptosis and anti-autophagy that contribute to the chronic infection of γHV68. Despite the discrepancies revealed in different experimental settings, the lack of absolute ablation of latency upon infection with the γHV68 vBcl-2 mutant suggests a complex nature of γHV68 persistence, involving multiple viral factors and cellular processes. Nevertheless, our studies on the role of vBcl-2, particularly its anti-autophagy function, in the maintenance of splenic latency highlight the importance of autophagy during γHV68 infection.

Despite the fact that vBcl-2 is primarily expressed during the γHV68 lytic cycle and that it efficiently blocks apoptosis in cell culture and in transgenic models [25],[26],[37], γHV68 vBcl-2 is dispensable for the initial evasion of apoptosis in acute infections, as also shown by Gangappa *et al.* and de Lima *et al.* [27],[37]. In this respect, one would expect that other anti-apoptotic lytic γHV68 genes be involved in the acute phase of γHV68 infections. Indeed, recent work by Feng *et al.* [43] indicates that γHV68 encodes a mitochondrion-associated anti-apoptotic protein (vMAP) that effectively antagonizes apoptosis and is required for the lytic replication of γHV68 in cell culture. It will then be of interest to test whether vMAP can also antagonize the host autophagy response and whether autophagy is involved in acute infections of γHV68. On the other hand, the preferential roles of vBcl-2 at the late stages of latent infection and ex vivo reactivation from latency strongly imply that its expression likely continues through into latent infection [27],[37]. This view is strengthened by the detection of the vBcl-2 transcript in latently infected cells and/or tissues, albeit in low abundance [41],[44],[45],[46]. However, it should be noted that because the vBcl-2-encoding M11 gene is interposed in the opposite orientation between the v-cyclin gene and LANA/ORF73, with its transcript overlapping with ORF73, it remains possible that the RT-PCR signal for vBcl-2 may correspond to an ORF73 mRNA encoded on the opposite strand. Due to the complex nature of transcription across this region, a strand specific transcript mapping may be necessary for clarifying the expression profile of vBcl-2 during different phases of viral infection in mice.

While dispensable for lytic replication, vBcl-2 has been previously indicated to be required for ex vivo reactivation [25],[37]. We further extended this view by showing that the reactivation efficiency of vBcl-2 correlates with its anti-apoptotic ability, not anti-autophagic effect. We found that a mutant strain of γHV68 that is specifically impaired in the apoptosis-inhibitory activity of vBcl-2, while remaining competent for autophagy inhibition, exhibited normal levels of splenic latency but inefficient ex vivo reactivation of the virus from the latently infected cells, suggesting that apoptosis evasion by vBcl-2 is particularly important during the reactivation process of the virus from latency. Notably, this viral phenotype, associated with the ΔBH2 mutant, was revealed at day 28 dpi but not at earlier time points of latency. This implies that the viral maintenance of latency represents a genetically distinct phase of γHVs infection and involves different viral and/or host factors. In this scenario, it is more likely that additional apoptosis inhibitors of γHV68 and/or the host proteins may be required for, or at least directly involved, for ex vivo reactivation in the early stage of latency, compensating for the anti-apoptotic effect of vBcl-2. Therefore, our studies do not preclude the importance of apoptosis regulation for ex vivo reactivation at the early stage of latency, but rather have identified a vBcl-2-associated deficit in latency maintenance. Such a deficit would also presumably reduce the virulence of γHV68. Furthermore, it has been set forth that the poor viability of explanted murine B cells may conceivably affect the efficiency of ex vivo reactivation. It is thus possible that the removal of the BH2 domain, which mitigates the anti-apoptotic properties of vBcl-2, may affect the survival of explanted B cells, thereby indirectly impacting the efficiency of the ex vivo reactivation of the virus. However, this possibility did not manifest itself in the early time points of viral latency, suggesting that survival of latently infected B cells in culture does not play a critical role in dictating the phenotype of the γHV68 vBcl-2 ΔBH2 mutant and that the vBcl-2-mediated inhibition of host apoptosis may be more directly involved in the reactivation programming of γHV68. Nevertheless, our study of the requirement of vBcl-2 for both latency maintenance and reactivation process is consistent with vBcl-2 being expressed in latently infected tissues [45]. It also points to the unique protective activities of vBcl-2 mutants in apoptosis and autophagy with respect to distinct phases of viral infection, and also their coordinated effects on γHV68 persistency and/or pathogenesis.

γHVs have developed a unique mode of interaction with the host where they establish lifelong latency and may be reactivated throughout the life of the host, which has been associated with the onset of various malignancies. Although it remains to be fully understood what factors govern the establishment and maintenance of latency, our study clearly demonstrates that sustained γHV68 latency in splenocytes requires the vBcl-2-mediated inhibition of the host autophagy machinery. But, how autophagy functions and what accounts for its effect are not yet understood. Given the substantial contributions of autophagy to the quality control of cytoplasmic components in host cell, most simply, autophagy induction may promote the degradation of cytosolic viral protein(s) essential for the maintenance of latency. Alternatively, the ‘autophagic digestion’ of viral latent antigens may facilitate its MHC class II presentation and cytotoxic T cells (CTL) recognition, as recently exemplified by the nuclear antigen 1 of Epstein-Barr virus (EBNA1) [47]. Additionally, autophagy may help to deliver a ‘viral signal’ to TLR-containing endosomes, thus stimulating the IFNα production that has been proven to be important to the control of acute γHV68 infection as well as latency [48],[49],[50],[51]. It is also possible that the autophagy induced when Beclin1 is unchecked by vBcl-2 can trigger cell death of latently infected cells, such a scenario is supported by the fact that a Beclin1 mutant unable to bind to Bcl-2 induces caspase-independent autophagic cell death [12]. The observations that autophagy may promote the sequestration and digestion of replicating viruses inside the host cell as described in HSV-1 [52],[53] could also provide an attractive explanation for the restriction of persistent infection of γHV68, but direct evidence is missing. While it is not yet clear by which mechanism(s) autophagy restricts viral persistency, none of these mechanisms are mutually exclusive and there may be other consequences of autophagy function relating to the activation of the host immune responses against γHV68 and latency, as well. Further studies examining the molecular details involved in the vBcl-2-mediated inhibition of autophagy will expand our understanding of both autophagy and γHV-associated pathogenesis and reveal novel targets for antiviral therapy.

In conclusion, we have described a crucial role for the viral evasion of autophagy in latent viral infections. Beyond its established anti-apoptotic functions, vBcl-2 targets the host autophagy effector protein Beclin1 and this activity of vBcl-2 is essential to the viral maintenance of latency. Our findings thus indicate that two host innate immune pathways, autophagy and apoptosis, both targeted by vBcl-2, actually conduct hitherto unexpected and distinctive roles in protecting against viral infections. Future studies will aim to analyze in detail the molecular mechanisms of autophagy that contributes to controlling γHV infection.

## Materials and Methods

### Mice

All mice handling was performed in accordance with the Animal Research Committee guidelines of the University of Southern California (USC) and the University of California, Los Angeles. All methods used herein have also been approved by the USC Animal Research Committee. BALB/c mice were obtained from Charles River Laboratories, Inc. (Wilmington, MA). All mice (∼6-week old, n = 5∼8 per pool) were infected intranasally with 5,000 plaque-forming units (PFU) of γHV68 viruses under brief halothane anesthesia and the infected mice were sacrificed at 5 and 7 days post-infection (dpi) to measure acute infection in the lungs or at 12 dpi, 14 dpi, 21 dpi, 28 dpi, 35 dpi, and 42 dpi to measure viral latent load in the spleen.

### Cell culture and viruses

NIH3T3, BHK21 and 293T cells were cultured in Dulbecco's modified Eagle's medium (DMEM) supplemented with 10% fetal bovine serum, 2 mM L-glutamine, and 1% penicillin-streptomycin (Gibco-BRL). Transient transfection was performed with Fugene 6 (Roche), Lipofectanine 2000 (Invitrogen), or Calcium phosphate (Clontech). NIH3T3 stable cell lines were established using a standard protocol of selection with 2 µg/ml of puromycin (Sigma-Aldrich). The wild-type (WT) γHV68 virus, pBAC/γHV68 virus [54], and its mutant derivatives were all propagated in BHK21 cells for in vitro studies and in NIH3T12 cells for in vivo studies.

### Plasmid construction

A DNA fragment corresponding to the γHV68 vBcl-2 coding sequence was amplified from S11 genomic DNA. The PCR-amplified vBcl-2 DNA was then cloned into a modified pEF-IRES-puro vector (Invitrogen) encoding an N-terminal HA tag (pEF-HA-vBcl-2). Mutations in the vBcl-2 gene were generated by PCR (Hi-Fidelity PCR kit, Roche) with oligonucleotide-directed mutagenesis. Specifically, vBcl-2 Δα1 (lacking the N-terminal 21 residues) and ΔTM (lacking the C-terminal 20 residues) deletion constructs were amplified from the pEF-HA-vBcl-2 vector using specific primers; The vBcl-2 ΔBH2 (lacking residues 129-144 of BH2 domain) and Δα7 [lacking residues 130–134 (NHFPL)] mutants were created via two-step PCR mutagenesis. The HA-vBcl-2 AAA mutant with alanine substitutions at the Ser85-Gly86-Arg87 residues was created using a Quickchange site-directed mutagenesis kit (Stratagene). All of the PCR products with the indicated vBcl-2 mutations were then cloned in frame into the *Xho*I/*Mlu*I sites of the pEF-IRES-puro vector, for both transient and stable expression. All mutant constructs were completely sequenced to ensure the presence of the desired mutation and the absence of secondary mutations. Constructs expressing the HA-tagged Bcl-2 family proteins were kindly provided by J. Marie Hardwick (John Hopkins university). The Beclin1-V5 plasmid has been described previously [29]. For yeast-two hybrid analyses, vBcl-2 and its mutant derivatives were cloned into the *Eco*RI/*Bam*HI sites of the yeast plasmid pGBKT7 (Clontech), which carries the *S. cerevisiae TRP1* gene as a selectable marker. The BH3-like domain (residues 88-150) of Beclin1 was subcloned into the *Eco*RI/*Xho*I sites of the pGADT7 vector (Clontech), harboring the *LEU* selection marker. To produce a GST fusion protein of Bak (GST-BakΔTM) from *E. coli*, the PCR product of Bak cDNA with a deletion of the C-terminal TM region was subcloned into the *Eco*RI/*Xho*I sites of pGEX4T-1. All constructs were sequenced using an ABI PRISM 377 automatic DNA sequencer.

### Viral mutagenesis

To make specific mutants of γHV68 (i.e. HA-WT, HA-Δα1, HA-ΔBH2, and HA-AAA), the two-step bacteriophage lambda Red-mediated homologous recombination method was performed using the γHV68 bacterial artificial chromosome (BAC) clone in GS1783 (an *E. coli* strain containing an arabinose-inducible I-SceI gene, provided by G. Smith, Northwestern University Medical School) as previously described [55]. Briefly, PCR was used to generate constructs containing the kanamycin-resistance (Kan^R^) gene with the mutated vBcl-2 gene. This kanamycin cassette was then inserted into the γHV68 BAC clone by homologous recombination and a markerless mutation was achieved through the deletion of the kanamycin resistance gene using I-SceI. Consequent mutations in the BAC DNAs were confirmed by DNA sequencing and the genomic integrity of the mutated BAC MHV-68 was investigated by restriction enzyme digestion and southern blot analysis as previously described [54]. vBcl-2-null BAC was generated by the in vitro MuA transposition of signature tagged transposon [54]. All BACs were reconstituted into infectious viruses by transfecting the BAC DNA along with the *Cre* recombinase-expressing plasmid, which removes BAC vector sequence, into NIH3T12 cells using Lipofectamine Plus reagent (Invitrogen). The produced viruses were purified as single clone by limiting dilution and then amplified in NIH3T12 cells. The purified viral stock was tittered by plaque assays, using a monolayer of Vero cells overlaid with 1% methylcellulose. By 5 days post-infection, the cells were fixed and stained with 2% crystal violet in 20% ethanol. Plaques were then counted to determine infectious titer.

### Yeast two-hybrid assay

To analyze the interactions between the Beclin1 and vBcl-2 mutants, yeast strain AH109, expressing the BH3-like domain of Beclin1 fused to the Gal4 activation domain in the pGADT7 plasmid, was used to transform pGBKT7 plasmids containing the mutants of vBcl-2, and the transformants then assayed for α-galactosidase activity, as previously described [56].

### Autophagy analyses

Quantitative GFP-LC3 light microscopy autophagy assays were performed in NIH3T3 stable cells expressing the WT or mutant forms of vBcl-2, then transfected with a GFP-LC3-expressing plasmid [35]. Autophagy was then induced by starvation or rapamycin treatment. For starvation, the cells were washed three times with PBS and incubated in Hank's solution (Invitrogen) for 4 h at 37°C. Alternatively, the cells were cultured in DMEM containing 1% FBS and 2 µM rapamycin (Sigma-Aldrich) for 6 h. LC3 mobility shift was detected by immunoblotting as previously described [29]. For autophagy levels during viral infection, NIH3T3 cells were transfected with GFP-LC3, then infected with recombinant γHV68 WT or mutant viruses at an MOI of 5, and fixed 18 h after infection.

### Apoptosis analyses

NIH3T3 cells stably expressing the WT or mutant forms of vBcl-2 were seeded at 1×10^6^ cells per well into 6-well plates for 24 h. The cells were then treated with fresh medium containing 2 ng/ml tumor necrosis factor alpha (TNFα) plus 1 µg/ml cycloheximide (CHX) for up to 12 h. For the cell viability assay, the cells were stained with trypan blue for dye exclusion. For the analysis of apoptotic cells, the samples were prepared using an *DEADEND™* Fluorometric TUNEL system kit (Promega) according to the manufacturer's instructions. Nuclei were counterstained with 4, 6-diamidino-2-phenylindole (DAPI). Fluorescence microscopy analyses were performed with an Olympus IX-70 microscope. The percentage of TUNEL-positive cells was determined against the number of DAPI-stained nuclei. For the PI staining assay, the cells were collected with the cell dissociation buffer (Sigma-Aldrich) and then fixed with 70% of ethanol overnight at −20°C. Fixed cells were washed twice with PBS, and incubated in PBS containing propidium (PI; 5 µg/ml), RNase A (1 mg/ml), and Triton X-100 (0.5%) at room temperature for 30 min. Fluorescence emitted from the propidium–DNA complex was measured using FACScan flow cytometry. Cells containing hypodiploid DNA were considered apoptotic. The data was analyzed using Cell Quest (BD Bioscience). For caspase-3 activity assay, the cells were harvested after treatment, washed three times with PBS and fixed with fixation medium (Invitrogen, Catalog# GAS001S) for 15 min, permeabilized with Permeabilization Medium (Invitrogen, Catalog# GAS002S) for another 15 min, and then stained with PE-conjugated anti-Caspase3 active form (BD biosciences #550821) for flow cytometry analysis. Data was analyzed by FlowJo-6.4. For apoptotic levels during viral infection, NIH3T3 cells were infected with recombinant γHV68 WT or mutant viruses at an MOI of 5, and apoptosis was assessed by TUNEL staining and nuclei counterstaining as described above.

### Growth curves

BHK21 cells and NIH3T3 cells were seeded at 2×10^5^ cells per well into 6-well plates for the single-step growth curve with a multiplicity of infection (MOI) of 5.0, or at 1×10^5^ cells per well for multi-step growth curves with an MOI of 0.1. The samples were harvested at various time points post-infection, subjected to three freeze-thaw cycles, then titered by plaque assay in triplicate as previously described [37].

### Lung titer and infectious center assay

To determine the virus titer in the infected lungs, the lungs were homogenized in 1 ml of DMEM and the infectious viruses in the homogenate supernatants was measured by three independent plaque assays. For infectious center assay, which measures the amount of the latent virus that is able to reactivate from the latently infected B cells, single cell suspensions of splenocytes were prepared from the infected spleens and co-cultivated with a monolayer of Vero cells overlaid with 1% methylcellulose. The Vero cells were incubated further for 5 days, then fixed and stained with 2% crystal violet in 20% ethanol. Plaques were then counted to determine the infectious centers [57]. A majority of the samples in the assay for preformed viruses resulted in no plaque, with a minority of samples displaying 1 to 2 plaques per ∼10^7^ splenocytes.

### Quantification of the viral genome

For quantification of viral genome load from the infected cells/tissues, total genomic DNA from the infected organs was prepared and subjected to quantitative real-time PCR, as previously described [54]. Briefly, total genomic DNA from the infected lungs or the spleen tissues was extracted using a DNeasy Tissue Kit (QIAGEN, Valenia, Calif.), according to manufacturer's instructions. γHV68 ORF56-specific primers (forward primer: 5′-GTAACTCGAGACTGAAACCTCGCAGAGGTCC-3′; reverse primer: 5′-CCGAAGCTTGCACGGTGCAATGTGTCACAG-3′) were used in the assay. The DNA templates were mixed with 2× Master mix (Biorad iQ™ SYBR® Green Supermix) and PCR was performed at 95°C for 15′ and 45 cycles of 95°C for 30″, 60°C for 30″, and 72°C for 30″, followed by melting curve analyses. 100–500 ng of DNA was analyzed in duplicate for each sample and compared with a standard curve of a BAC plasmid containing the γHV68 genome, serially diluted with uninfected cellular DNAs and amplified in parallel. Amplification and detection were performed using Opticon II (MJ Research). The specificity of the amplified products was confirmed by agarose gel electrophoresis.

Quantitative analyses of v-cyclin transcript were performed using SYBR GreenER™ qPCR Kit (Qiagen) on a DNA Engine Opticon® 2 continuous Fluorescence Detection System (MJ Research, Incorporated, Waltham, MA). Total RNA was extracted from the infected cells using Trizol (Invitrogen) and 100 ng of purified total RNA was reverse transcribed to cDNA using a cDNA synthesis kit (Invitrogen). The PCR reaction was set according to the manufacturer's recommendations. Briefly, after an initial 5 minutes of denaturation at 95°C, thermal cycling was performed at 94°C for 45″, 57°C for 1′, and 72°C for 1′ for a total of 40 cycles followed by a melting curve analyses. The amount of RNA was normalized with the quantified β-Actin in each sample. The primer sets for amplification of orf72 were: forward, 5′-GGAGCAACAACAGCTGACAA-3′; reverse, 5′-GTGATTAGCACTGGGCGTTT-3′. The primer sets for β-Actin were: forward, 5′-CGAGGCCCAGAGCAAGAGAG-3′; reverse, 5′-CGGTTGGCCTTAGGGTTCAG-3′. Quantitative experiments were performed at least three times, including a no-template control each time. The size of the amplified products was confirmed by agarose gel electrophoresis.

### Immunoblotting, immunoprecipitation and GST pull-down

For immunoblotting, the polypeptides were resolved by SDS-PAGE and transferred onto a PVDF membrane (Bio-Rad). The membranes were blocked with 5% non-fat milk, and probed with the indicated antibodies. Goat antibodies coupled to horseradish peroxidase specific to mouse or rabbit immunoglobulins were used as secondary antibodies (diluted 1∶10,000, Sigma-Aldrich). Immunodetection was achieved with a chemiluminescence reagent (Pierce) and detected by a Fuji Phosphor Imager (BAS-1500; Fuji Film Co., Tokyo, Japan).

For immunoprecipitation, cells were harvested and then lysed in a 1% NP40 lysis buffer supplemented with complete protease inhibitor cocktail (Roche). After pre-clearing with protein A/G agarose beads for 1 h at 4°C, whole-cell lysates were used for immunoprecipitation with the indicated antibodies. Generally, 1–4 µg of the commercial antibodies was added to 1 ml of the cell lysate, which was then incubated at 4°C for 8–12 h. After addition of protein A/G agarose beads, incubation was continued for another 2 h. Immunoprecipitates were extensively washed with an NP40 lysis buffer and eluted with an SDS-PAGE loading buffer by boiling for 5 min.

For in vitro GST pull-down assay, GST by itself or a GST-BakΔTM fusion protein was purified from *E.coli* strain BL21 (DE3) (Promega). 293T cell lysates were incubated with glutathione beads containing the GST fusion protein in a binding buffer (20 mM HEPES [pH 7.4], 100 mM NaCl, 1% NP-40, and protease inhibitors) for 2 h at 4°C. The glutathione beads were then washed four times with the binding buffer, and the proteins associated with the beads were analyzed by SDS-PAGE and subjected to immunoblot assay with the phosphorimager.

### Immunofluorescence and confocal laser scanning microscopy

NIH3T3 stable cells grown on 8-well chamber slides were fixed with 2% (w/v) paraformaldehyde in PBS for 20 min, permeabilised with 0.2% (v/v) Triton X-100 for 15 min and blocked with 10% goat serum (Gibco-BRL) for 1 h. Primary antibody staining was performed using antiserum or purified antibodies in 1% goat serum for 1–2 h at room temperature. The cells were then extensively washed with PBS and incubated with diluted secondary antibodies in 1% goat serum for 1 h. The cells were mounted using Vectashield (Vector Laboratories, Inc.). The confocal images were acquired using a Leica TCS SP laser-scanning microscope (Leica Microsystems, PA) fitted with a 100× Leica objective (PL APO, 1.4NA) and Leica image software.

### Statistical analysis

Statistical analyses were performed using unpaired *t*-tests. Values are expressed as mean±SEM of at least three independent experiments unless otherwise noted. A *P* value of ≤0.05 was considered statistically significant.

## Supporting Information

Figure S1 (A) NIH3T3 cells stably expressing WT or mutant forms of vBcl-2 were transfected with GFP-LC3, then incubated under normal or starvation conditions for 4 h. The number of GFP-LC3-positive dots per cell was counted using a fluorescence microscope. Data represents mean±SEM of the combined results from three independent experiments. **, *P*<0.0001. (B) The expression of WT and mutant vBcl-2 proteins in NIH3T3 cells was determined by immunoblotting using an anti-HA antibody. β-actin was probed as a loading control. (C) Intracellular localization of vBcl-2 WT and its mutants in NIH3T3 cells. NIH3T3 cells stably expressing HA-tagged WT vBcl-2 and its mutants were fixed and the localization of vBcl-2 proteins was determined by staining with an anti-HA antibody using confocal microscopy. Scale bar, 5 µm. (D) Restriction enzyme digestion patterns (top) and Southern blot analysis (bottom) of wild-type (wt) γHV68 and vBcl-2 mutants. Bacterial artificial chromosome (BAC) DNAs of wt and mutants were prepared and digested with *BamH*I, *EcoR*I, or *Hind*III. The digested DNAs were resolved by 1% agarose gel electrophoresis. M: 1 Kb DNA Ladder (Invitrogen); WT: wild-type γHV68; Null: vBcl-2 null mutant by transpon-insertion. The asterisk (*) indicates the heterogeneity of the 40-bp repeat in the vBcl-2AAA mutant, which often occurs in our BAC system but does not affect viral replication both in vitro and in vivo [57]. The enzyme digested DNAs were transferred to nitrocellulose membrane and hybridized with ^32^P-labeled probes for M11 gene and transposon. Expected sizes (bp) for *Bam*HI digests: wt γHV68, 5249 bp; null mutant, 3473, 2060, and 1033 bp; HA-WT γHV68, 5288 kb; HA-vBcl-2AAA mutant, 5288 bp; HA-vBcl-2Δα1 mutant, 5228 bp; HA-vBcl-2ΔBH2 mutant, 5240 bp. Expected sizes (bp) for *Eco*RI digests: wt γHV68, 5186 bp and 3147 bp; null mutant, 4881, 3147, and 1622 bp; HA-WT γHV68, 5225 bp and 3147 bp; HA-vBcl-2AAA mutant, 5225 bp and 3147 bp; HA-vBcl-2Δα1 mutant, 5165 bp and 3147 bp; HA-vBcl-2ΔBH2 mutant, 5177 bp and 3147 bp. Expected sizes (bp) for *Hind*III digests: wt γHV68, 8357 bp and 2882 bp; null mutant, 8401, 2882, and 1273 bp; HA-WT γHV68, 8396 bp and 2882 bp; HA-vBcl-2AAA mutant, 8396 bp and 2882 bp; HA-vBcl-2Δα1 mutant, 8336 bp and 2882 bp; HA-vBcl-2ΔBH2 mutant, 8348 bp and 2882 bp. The 3147 bp and 2882 bp bands for the *Eco*RI and *Hind*III digests are derived from the fragment of the BAC vector that hybridizes to the probe for M11 gene, because the probe was generated by the random hexamer labeling of a plasmid containing vBcl-2 gene.
(5.19 MB TIF)

Figure S2 (A) Immunoblot analysis of vBcl-2 expression in NIH3T3 cells infected with the WT γHV68 virus (lane 1), recombinant γHV68 expressing HA-tagged WT vBcl-2 (lane 2), Δα1 (lane 3), AAA (lane 4), or ΔBH2 (lane 5) mutants. β-actin was probed as a loading control. (B) Transcription of v-cyclin in recombinant γHV68-infected cells in culture. NIH3T3 cells were infected with the WT or recombinant γHV68 viruses expressing the indicated vBcl-2 constructs. Total RNA was extracted from the infected cells and the β-actin normalized v-cyclin expression was quantified by real-time RT-PCR (bottom) with products of the reaction electrophoresed in a 2% agarose gel (top). Negative control (negative ct) indicates reaction from the mock-infected NIH3T3 cells. Samples were run in triplicate and the data are representative of three independent experiments.
(4.43 MB TIF)

Figure S3 Anti-apoptotic Activities of the vBcl-2 Mutant Proteins. NIH3T3 cells stably expressing the WT or mutant forms of vBcl-2 were treated with TNFα and cycloheximide (CHX) for 12 h, then assayed for PI staining followed by flow cytometry analysis. Apoptosis was quantified as mean±SEM of the combined results from three independent experiments. PI, propidium iodide.
(2.30 MB TIF)

Figure S4 Single-step (bottom) and multiple-step (up) growth curves of WT and recombinant γHV68 viruses in NIH3T3 cells.
(2.02 MB TIF)

Figure S5 (A, B, C, D) BALB/c mice were infected intranasally with the WT virus, an independent isolate of the Δα1 (Δα1-IND) mutant, or of the ΔBH2 (ΔBH2-IND) mutant γHV68. Viral genome loads were then measured by real-time PCR at 7 dpi (A) in the lungs, at 14 dpi (B) and 28 dpi (D) in the spleens. Splenic infectious centers (C) were also measured at 28 dpi in the spleens. Values are mean±SEM. n.s., not significant. *, *P*<0.05; **, *P*<0.01; ***, *P*<0.001. (E) The number of splenocytes of the WT or mutant vBcl-2 γHV68 infected mice at 28 dpi.
(4.95 MB TIF)
