# Supplementary figures and images for: Viral Bcl-2-Mediated Evasion of Autophagy Aids Chronic Infection of γHerpesvirus 68
Source: PLoS Pathog. 2009 Oct 9;5(10):e1000609. doi: 10.1371/journal.ppat.1000609 (PMC2752191; doi:10.1371/journal.ppat.1000609)

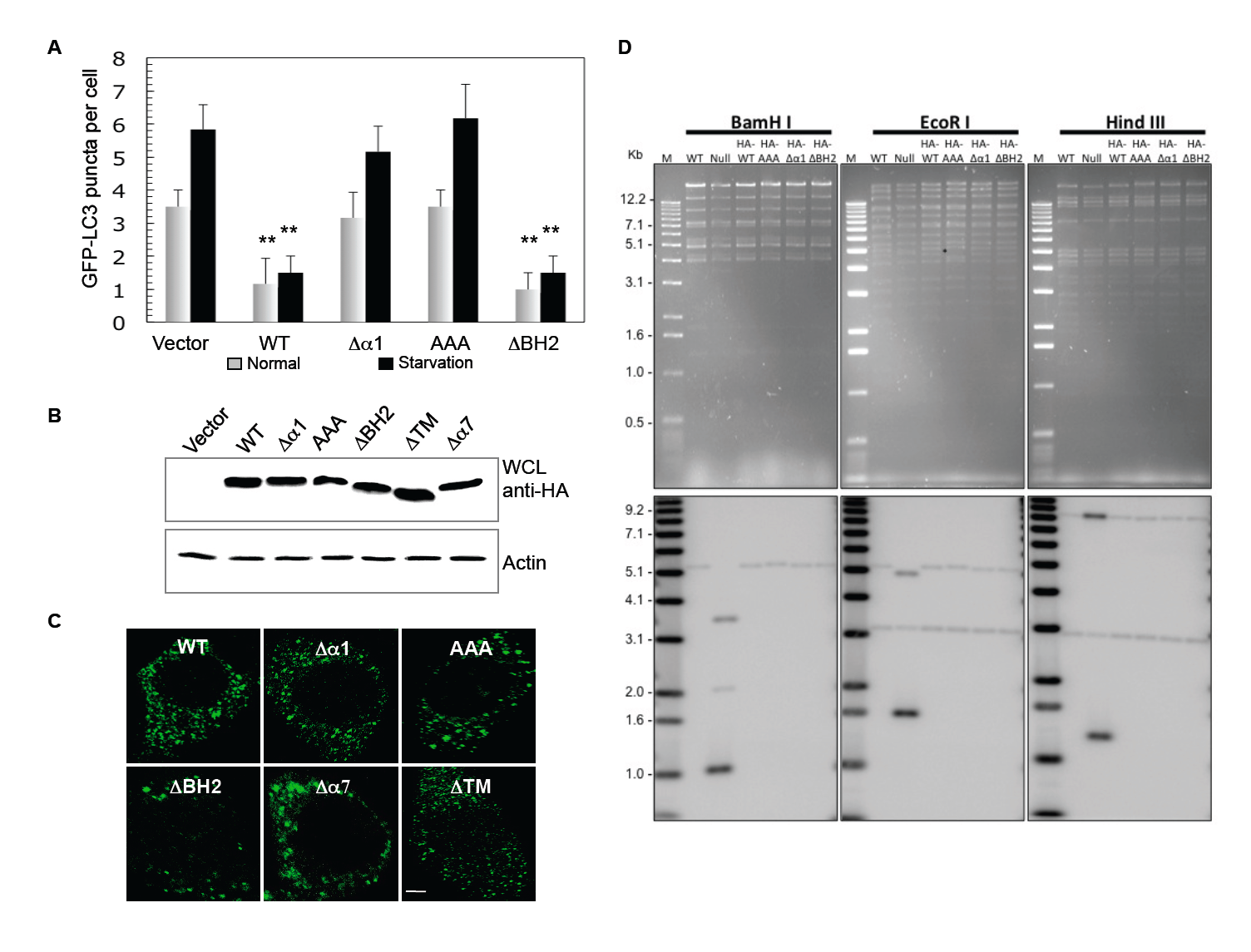

Supplement: Figure S1 — (A) NIH3T3 cells stably expressing WT or mutant forms of vBcl-2 were transfected with GFP-LC3, then incubated under normal or starvation conditions for 4 h. The number of GFP-LC3-positive dots per cell was counted using a fluorescence microscope. Data represents mean±SEM of the combined results from three independent experiments. **, P<0.0001. (B) The expression of WT and mutant vBcl-2 proteins in NIH3T3 cells was determined by immunoblotting using an anti-HA antibody. β-actin was probed as a loading control. (C) Intracellular localization of vBcl-2 WT and its mutants in NIH3T3 cells. NIH3T3 cells stably expressing HA-tagged WT vBcl-2 and its mutants were fixed and the localization of vBcl-2 proteins was determined by staining with an anti-HA antibody using confocal microscopy. Scale bar, 5 µm. (D) Restriction enzyme digestion patterns (top) and Southern blot analysis (bottom) of wild-type (wt) γHV68 and vBcl-2 mutants. Bacterial artificial chromosome (BAC) DNAs of wt and mutants were prepared and digested with BamHI, EcoRI, or HindIII. The digested DNAs were resolved by 1% agarose gel electrophoresis. M: 1 Kb DNA Ladder (Invitrogen); WT: wild-type γHV68; Null: vBcl-2 null mutant by transpon-insertion. The asterisk (*) indicates the heterogeneity of the 40-bp repeat in the vBcl-2AAA mutant, which often occurs in our BAC system but does not affect viral replication both in vitro and in vivo [57]. The enzyme digested DNAs were transferred to nitrocellulose membrane and hybridized with 32P-labeled probes for M11 gene and transposon. Expected sizes (bp) for BamHI digests: wt γHV68, 5249 bp; null mutant, 3473, 2060, and 1033 bp; HA-WT γHV68, 5288 kb; HA-vBcl-2AAA mutant, 5288 bp; HA-vBcl-2Δα1 mutant, 5228 bp; HA-vBcl-2ΔBH2 mutant, 5240 bp. Expected sizes (bp) for EcoRI digests: wt γHV68, 5186 bp and 3147 bp; null mutant, 4881, 3147, and 1622 bp; HA-WT γHV68, 5225 bp and 3147 bp; HA-vBcl-2AAA mutant, 5225 bp and 3147 bp; HA-vBcl-2Δα1 mutant, 5165 bp and 3147 bp; HA-vBcl-2 [file ppat.1000609.s001.tif]

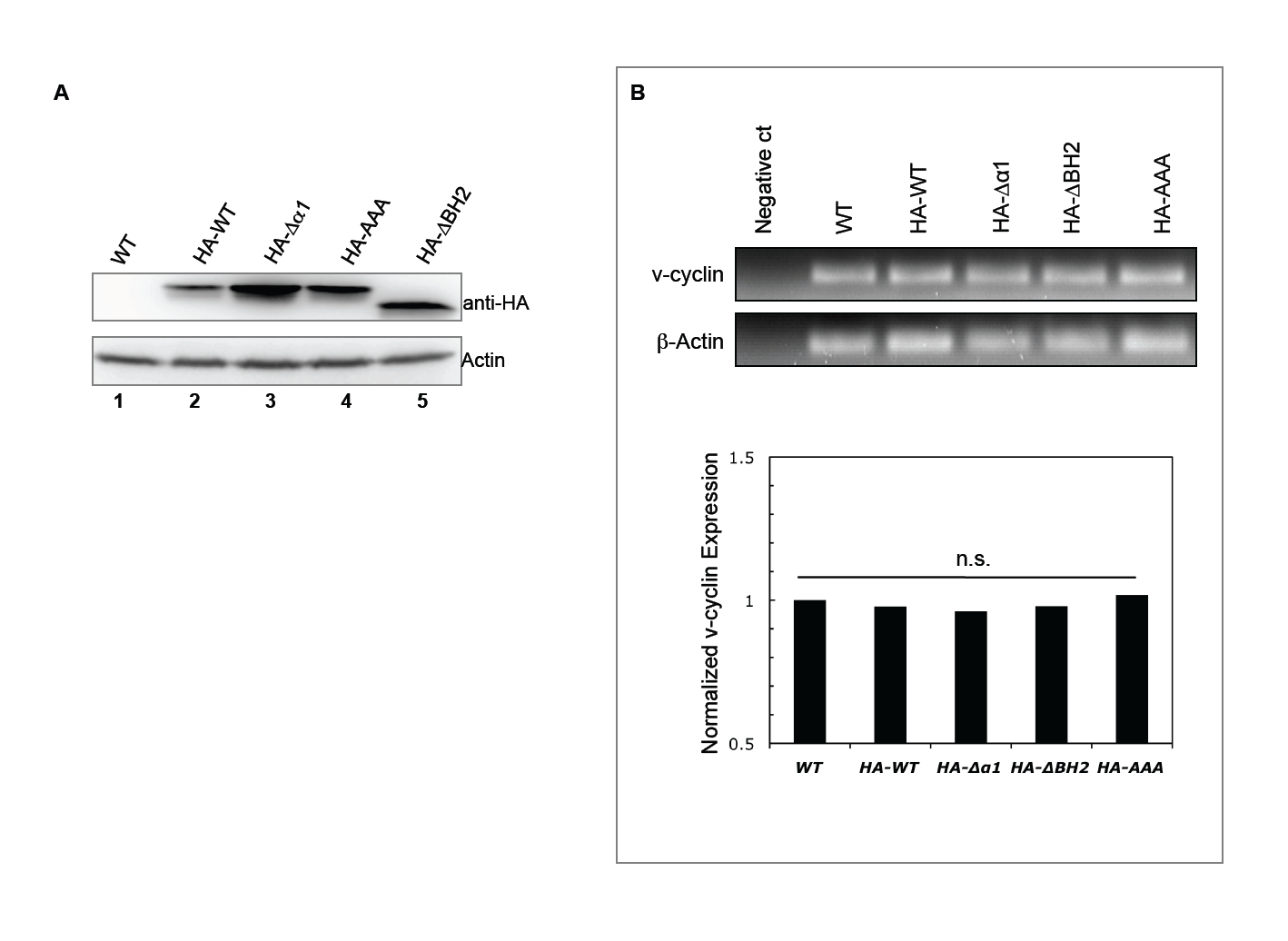

Supplement: Figure S2 — (A) Immunoblot analysis of vBcl-2 expression in NIH3T3 cells infected with the WT γHV68 virus (lane 1), recombinant γHV68 expressing HA-tagged WT vBcl-2 (lane 2), Δα1 (lane 3), AAA (lane 4), or ΔBH2 (lane 5) mutants. β-actin was probed as a loading control. (B) Transcription of v-cyclin in recombinant γHV68-infected cells in culture. NIH3T3 cells were infected with the WT or recombinant γHV68 viruses expressing the indicated vBcl-2 constructs. Total RNA was extracted from the infected cells and the β-actin normalized v-cyclin expression was quantified by real-time RT-PCR (bottom) with products of the reaction electrophoresed in a 2% agarose gel (top). Negative control (negative ct) indicates reaction from the mock-infected NIH3T3 cells. Samples were run in triplicate and the data are representative of three independent experiments. (4.43 MB TIF) [file ppat.1000609.s002.tif]

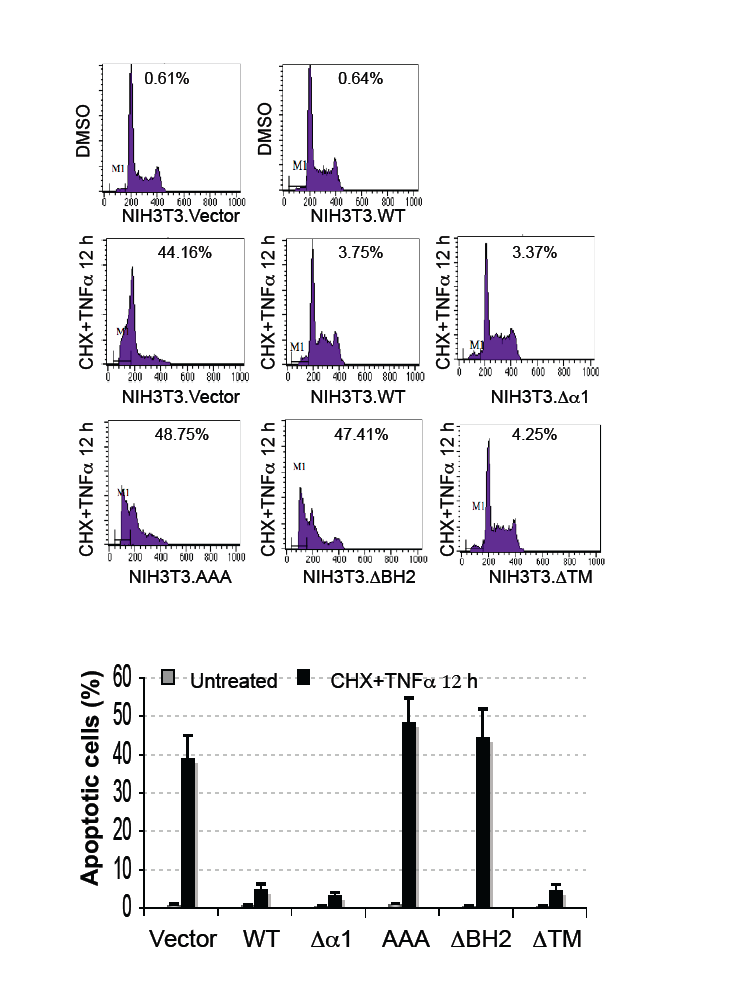

Supplement: Figure S3 — Anti-apoptotic Activities of the vBcl-2 Mutant Proteins. NIH3T3 cells stably expressing the WT or mutant forms of vBcl-2 were treated with TNFα and cycloheximide (CHX) for 12 h, then assayed for PI staining followed by flow cytometry analysis. Apoptosis was quantified as mean±SEM of the combined results from three independent experiments. PI, propidium iodide. (2.30 MB TIF) [file ppat.1000609.s003.tif]

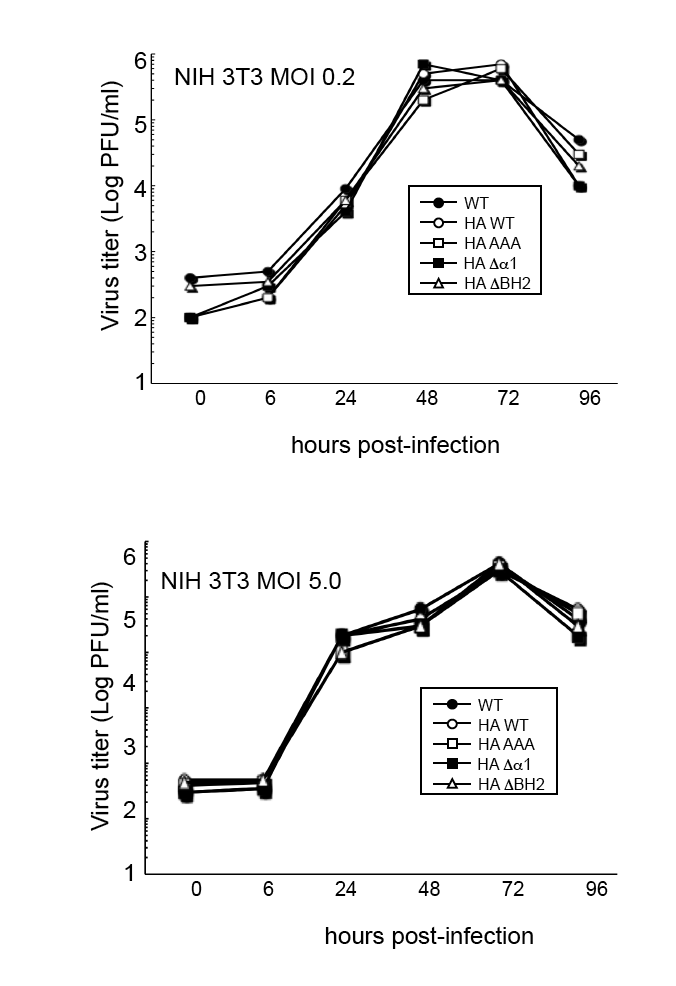

Supplement: Figure S4 — Single-step (bottom) and multiple-step (up) growth curves of WT and recombinant γHV68 viruses in NIH3T3 cells. (2.02 MB TIF) [file ppat.1000609.s004.tif]

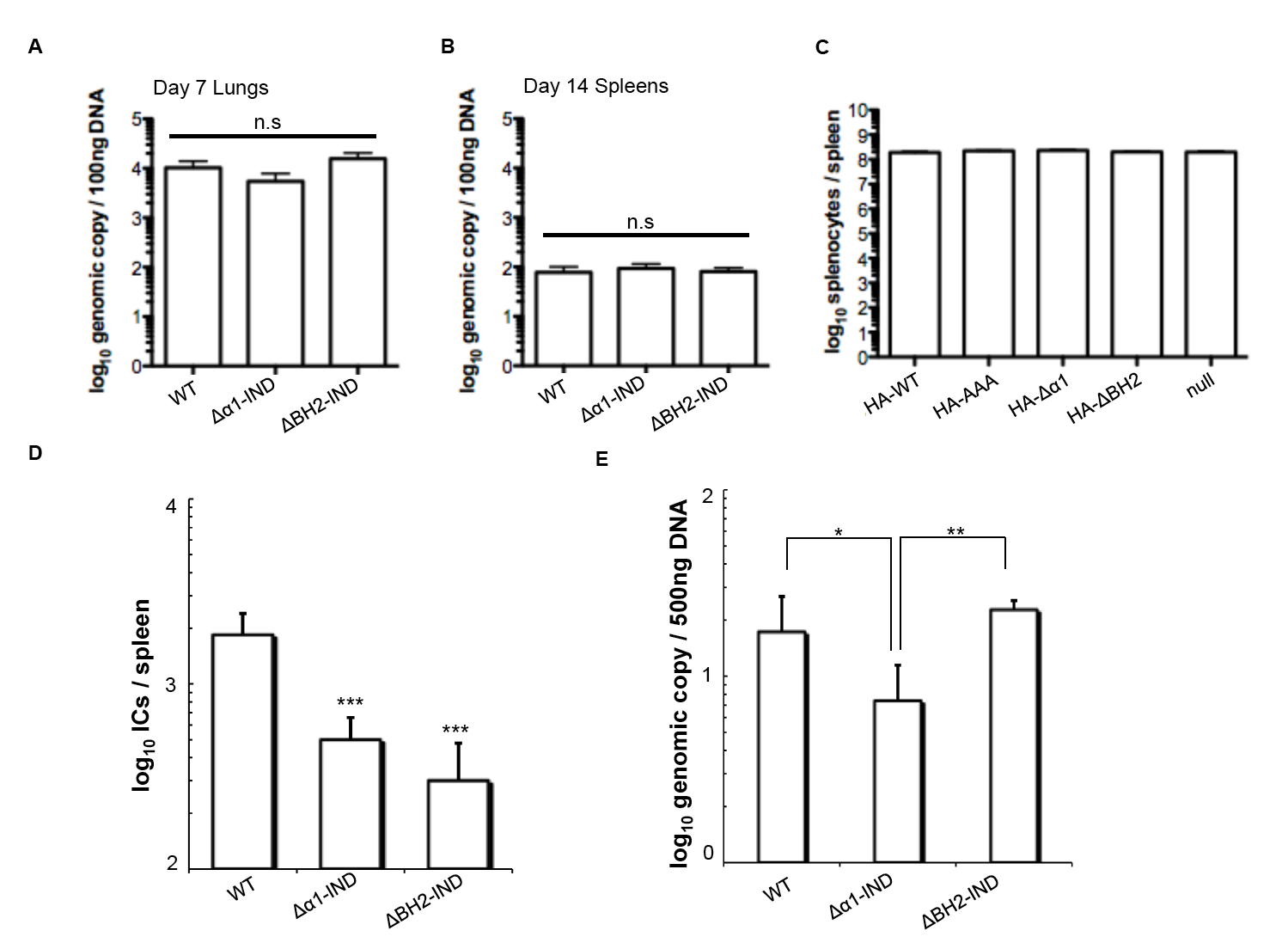

Supplement: Figure S5 — (A, B, C, D) BALB/c mice were infected intranasally with the WT virus, an independent isolate of the Δα1 (Δα1-IND) mutant, or of the ΔBH2 (ΔBH2-IND) mutant γHV68. Viral genome loads were then measured by real-time PCR at 7 dpi (A) in the lungs, at 14 dpi (B) and 28 dpi (D) in the spleens. Splenic infectious centers (C) were also measured at 28 dpi in the spleens. Values are mean±SEM. n.s., not significant. *, P<0.05; **, P<0.01; ***, P<0.001. (E) The number of splenocytes of the WT or mutant vBcl-2 γHV68 infected mice at 28 dpi. (4.95 MB TIF) [file ppat.1000609.s005.tif]
